# Particulate matters (PM_2.5_, PM_10_) and the risk of depression among middle-aged and older population: analysis of the Korean Longitudinal Study of Aging (KLoSA), 2016–2020 in South Korea

**DOI:** 10.1186/s12940-023-01043-1

**Published:** 2024-01-03

**Authors:** Hyunkyung Park, Cinoo Kang, AiMS-CREATE Team, Whanhee Lee, Insung Song, Dohoon Kwon, Jieun Oh, Jeongmin Moon, Jinah Park, Jieun Min, Ejin Kim, Hyemin Jang, Ho Kim

**Affiliations:** 1 Department of Public Health Sciences, Graduate School of Public Health, Seoul National University, 1 Gwanak-Ro, Gwanak-Gu, Seoul, 08826 Republic of Korea; 2 National Evidence-Based Health Care Collaborating Agency, 400 Neungdong-Ro, Gwangjin-Gu, Seoul, 04933 Republic of Korea; 3 School of Biomedical Convergence Engineering, College of Information and Biomedical Engineering, Pusan National University, 49 Busandaehak-Ro, Mulgeum-Eup, Yangsan-Si, Gyeongsangnam-Do 50612 South Korea; 4 Department of Environmental Medicine, College of Medicine, Ewha Womans University, 25 Magokdong-Ro 2-Gil, Ganseo-Gu, Seoul, 07804 Republic of Korea; 5 Institute of Sustainable Development, Seoul National University, 1 Gwanak-Ro, Gwanak-Gu, Seoul, 08826 Republic of Korea

**Keywords:** Air pollution, PM_2.5_, PM_10_, Older Korean adults, Depression

## Abstract

**Background:**

There is a growing concern that particulate matter (PM) such as PM_2.5_ and PM_10_ has contributed to exacerbating psychological disorders, particularly depression. However, little is known about the roles of these air pollutants on depression in elderly. Therefore, this study aimed to examine the association between PM_2.5_ and PM_10_, and depression in the elderly population in South Korea.

**Methods:**

We used panel survey data, the Korean Longitudinal Study of Aging (KLoSA), administered by the Labor Institute during the study period of 2016, 2018, and 2020 covering 217 districts in South Korea (*n* = 7674). Annual district-specific PM_2.5_ and PM_10_ concentrations were calculated for the study period from the monthly prediction concentrations produced by a machine-learning-based ensemble model (cross-validated R^2^: 0.87), then linked to the people matching with year and their residential district. We constructed a generalized estimating equation (GEE) model with a logit link to identify the associations between each of the long-term PM_2.5_ and PM_10_ exposures and depression (CES-D 10) after adjusting for individual and regional factors as confounders.

**Results:**

In single-pollutant models, we found that long-term 10 $\mathrm{\mu g}/{m}^{3}$ increments in PM_2.5_ (OR 1.36, 95% CI 1.20–1.56) and PM_10_ (OR 1.19, 95% CI 1.10–1.29) were associated with an increased risk of depression in the elderly. Associations were consistent after adjusting for other air pollutants (NO_2_ and O_3_) in two-pollutant models. In addition, the impacts substantially differed by regions grouped by the tertile of the population density, for which the risks of particulate matters on depression were substantial in the middle- or high-population-density areas in contrast to the low-population-density areas.

**Conclusions:**

Long-term exposure to PM_2.5_ and PM_10_ was associated with a higher risk of developing depression in elderly people. The impact was modified by the population density level of the region where they reside.

**Supplementary Information:**

The online version contains supplementary material available at 10.1186/s12940-023-01043-1.

## Introduction

Depression has been considered as a serious health problem worldwide and is also known that depression is an important factor exacerbating diseases such as sleep disorders, anxiety, loss of concentration, and chronic diseases [1, 2]. Globally, 264 million people are estimated to have depression in 2017 [3], and in South Korea, prevalence of depression has been increased from 3.72% in 2005 to 5.35% in 2013, as well as the consistent increase in number of patients visiting the hospital by depression from 2017 to 2021 [4, 5]. In addition, severe depression is reported as a crucial factor resulting in suicide, therefore, it is important to produce evidences on factors influencing depression and supporting policymakers to establish mental health-related intervention strategies based on quantitative assessment for risk factors, as South Korea is one of the countries recording the highest suicide rate [5, 6].

As an environmental factor, air pollution has been found to be associated with various health outcomes, including chronic disease and mental health [7–9]. In particular, particulate matters with diameter less than 2.5 $\mathrm{\mu m}$ (PM_2.5_) and diameter less than 10 $\mathrm{\mu m}$ (PM_10_) have been reported for its adverse effect on human health, including respiratory diseases, cardiovascular diseases, diabetes, and neuropsychological outcomes such as depression [10, 11]. Three explanations were introduced to understand the mechanism of depression: first, particulate matter enters the bloodstream and travels to the brain, where it causes neuroinflammation and oxidative stress, resulting in sadness [12, 13]. Second, particulate matter inhibits physical activity, which is reported to be associated with depression [14, 15], as well as social interaction. Third, with regard to the aging-related progression in the elderly population, it has also been suggested that aging promotes peripheral immune responses, which alter peripheral-brain immunological communication, resulting in the generation of pro-inflammatory cytokines that could exacerbate depression symptoms [16].

In a previous systematic literature review, it was reported that long-term exposure to PM_2.5_ was related to depression and anxiety in the general population [17]. Nevertheless, the evidence of an association between depression and air pollution in older people is still limited. There were studies investigating the relationship between air pollution and depression in the elderly population in some regions, such as South Korea [18–21], China [15, 22–24], the United States [25–27], Germany [28], and Taiwan [29]; however, the results were not consistent because some studies showed no significant association between particulate matter and depression in the elderly population [25, 26, 29]. Moreover, there is still a lack of evidence on whether the association between air pollution and depression differs by regional characteristics, such as urbanization levels, due to the limited number of studies covering both urban and rural areas. However, to the best of our knowledge, previous studies in South Korea only targeted the subjects who resided in highly urbanized areas near the capital city and metropolitan cities [18–21], therefore, there was an apparent limitation in examining whether the impacts of air pollution on depression in the elderly differ by regional characteristics, for instance, population density.

To address these gaps in knowledge, this study aimed to investigate the impacts of air pollution, particularly PM_2.5_ and PM_10_ concentrations, on depression in elderly people in South Korea using longitudinal data covering the majority areas of the country from 2016 to 2020.

## Materials and methods

### Study population

We obtained the data from the Korean Longitudinal Study of Aging (KLoSA) conducted by the Ministry of Employment and the Korea Employment Information Service (KESIS), which consisted of a nationwide sampled community-dwelling population aged 45 years or older in South Korea. KLoSA is a panel survey following up 10,254 nationwide participants randomly selected within stratified districts, those aged 45 years or older in 2006, and 920 participants were registered more in 2014. The panel study is a longitudinal study, similar to the cohort study. However, the difference is that the cohort study is based on a group of people with a common characteristic or who experienced the same life event during a given time period, whereas the panel study utilizes the same individuals as the sample over time. Since the panel study tracks the exact same respondents repeatedly, it allows the researcher to examine the exact changes in the same respondents that have taken place over time [30]. The KLoSA survey has collected individual information through questionnaire on demographic and socioeconomic characteristics, family relationships, mental health status, physical conditions, and residential area, and performed by every two years from the first survey in 2006 to the eighth in 2020. In this analysis, we used the data in 2016, 2018, and 2020 because PM_2.5_ monitoring stations have first been operated since 2015. The total number of the survey participants was 7,490, 6,940, and 6,488, along with a retention rate of 79.6%, 78.8%, and 78.1% for each year, respectively. For detailed information on the survey, KLoSA website (https://survey.keis.or.kr/eng/klosa/klosa01.jsp) provides a comprehensive protocol for the entire investigation process. Finally, in this study, we obtained a total of 7,674 study participants, with 7,479, 6,871, and 6,481 during the study periods of 2016, 2018, and 2020, respectively, after excluding participants who had missing values in depression measurement and covariates, or were lost to follow-up. A flowchart illustrating the inclusion and exclusion processes of participants in this study was displayed in the Additional file 1: Appendix Figure A.1. The study population resided in 217 districts, covering the majority of areas of South Korea.

### CES-D 10 (Center for Epidemiological Studies of Depression Scale)

The level of depression was measured using a CES-D 10, which is the widely used questionnaire to quantify depressive status [31–33]. The CES-D 10 is a short-form version of the CES-D questionnaire, consisted of 10 items to assess the depressive symptom levels during the past week; three items on depressed affect, two items on positive affect, three items on somatic symptoms, and two items on interpersonal relationships. Detailed questions included, “People were unfriendly.”, “I felt sad.”, “I felt depressed.”, “I felt like everything I did was an effort.”, “I felt that I was just as good as other people.”, “I felt that people disliked me.”, “My sleep was restless.”, “I enjoyed life.”, “I felt lonely.” and “I could not get going.”. Every item is based on a 4-point Likert scale (1: “rarely or none of the time [less than 1 day]”; 2: “Some or a little of the time [1–2 days]”; 3: “Occasionally or a moderate amount of the time [3–4 days]”; 4: “Most or all of the time [5–7 days]”). Likert scales for items 5 and 8 were reversely coded for the score. The final CES-D 10 score was calculated by summing up all scores of the 10 items, having a range from a minimum of 10 to a maximum of 40 indicating a higher score for higher severity of the depressive symptom. Cronbach's alpha for internal consistency was over 0.80 during the study period from 2016 to 2020.

### Air pollution data

We used monthly concentration predictions for ambient levels of PM_2.5_ and PM_10_ at 1 km^2^ spatial resolution across 226 districts in contiguous South Korea from a machine-learning-based validated model with high prediction performance (cross-validated R^2^: 0.87). This modeled air pollution concentration has also been utilized in a previous study [34]. This model is an ensemble model incorporating three machine learning algorithms: random forest regression, gradient boosting, and neural networks. Details of the prediction model are provided in the supplementary material (please see the Additional file 1: Appendix, 2. Air Pollution Prediction Model and Additional file 1: Figure A.2). From this data, we calculated the annual mean concentration of PM_2.5_ and PM_10_ for each of the 217 districts by averaging concentrations of grids for which the centroid point is inside the district boundary. Despite our capability to utilize finer-resolution air pollution data, the finest spatial resolution we could allocate for the study subjects was a district-level concentration because the KLoSA dataset only provided district-level information for the participants’ residences, rather than detailed home addresses. Annual district-specific air pollution concentrations for the years 2016, 2018, and 2020 are then linked to the individuals, representing long-term air pollution exposure levels corresponding to their residential district each year. We also calculated annual concentrations of NO_2_, 8-h maximum O_3_ (cross-validated R^2^: 0.76 and 0.84, respectively) to utilize other pollutants as confounders to identify the PM_10_ and PM_2.5_ impacts after controlling for them.

### Covariates

The KLoSA survey includes questions about a wide array of socioeconomic characteristic and physical condition. We considered ten individual-level variables including sex (male/female), age group (53–64, 65–74, 75–84, and ≥ 85 years), current smoking (no/yes), current drinking (no/yes), education attainment (primary school or less/middle and high school/college or higher), marital status (single [single, divorced, and widowed, married and not living with spouse]/married [married and living with spouse]), social contact (< 1/month [every other month or less often], > 1/month and < 1/week [once a month or more often and less than once a week], and > 1/week [once a week or more often]), self-reported health status (good [good or better]/not good [normal or bad]), exercise (no/yes), private medical insurance (no/yes). We also considered regional characteristics including population density, number of beds in hospitals per 1,000 persons, number of national basic livelihood beneficiaries, independent rate of finance of local government, and the proportion of basic pension beneficiaries. The population density was classified by the tertiles of the population density distribution (low, mid, and high: < 161, 161–3591, and ≥ 3591 people/${km}^{2}$, respectively), and other regional variables were considered on the continuous scale. The regional characteristics information was based on 2016, and the data were obtained from the Korea Statistical Information Service (http://kosis.kr).

### Statistical analysis

Descriptive statistics for individual characteristics of the study population were presented with a number of participants and proportion for categorical variables, mean and standard deviation (SD) for continuous variables. Summary statistics of air pollution concentrations (PM_2.5_ and PM_10_) and CES-D 10 scores were presented by each of the three KLoSA panel years (2016, 2018, and 2020). We constructed a generalized estimating equation (GEE) model to identify the associations between each of the long-term PM_2.5_ and PM_10_ exposure (per 10 $\mathrm{\mu g}/{m}^{3}$ increment) and depression. The KloSA panel data has been collected biennially (i.e., 2-year-interval) follow-up, thus the exact time of the outcome occurrence within a wide range of the 2-year period could not be adequately captured, and the follow-up time of this study is also relatively short. In addition, the odds ratio of depression associated with air pollution would be more interpretable from an epidemiological perspective. Thus, we used GEE in this study, which has been developed to analyze repeatedly measured longitudinal data using a binary-scale outcome, and can account for within-subject variability and consider changes in outcome status over time in the model. CES-D 10 score was converted into a binary variable as the dependent variable for depression, with 20 points and above, or less than 20 points, which has been suggested as the optimal cut-off point for defining depression [35–37].

First, we performed the crude model that only adjusted for baseline depression status. Second, the model was additionally adjusted for sex, age group, current smoking, current drinking, education attainment, marital status, social contact, self-reported health status, exercise, private medical insurance, longitude, latitude, the interaction term of the longitude and latitude, population density, number of beds in hospitals per 1,000 persons, number of national basic livelihood beneficiaries, independent rate of finance of local government, and the proportion of basic pension beneficiaries (main models). The longitude, latitude, and interaction term of the longitude and latitude were included to account for regional variation in depression. Third, we performed the two-exposure models, which were additionally adjusted for each of the other air pollutants such as NO_2_ and O_3_ from the main models, to determine if the particulate matter has an effect even when other air pollutants are present. Fourth, in the main models, we replaced the particulate matter concentrations from the calendar year-based one-year average concentrations with the averaged 12-month concentrations before the actual survey date (i.e., an average concentration from September of the previous year to August of the current year in 2016, 2018, and August of the previous year to July of the current year in 2020), to account for potential exposure misclassification bias. Fifth, we considered depression as a continuous rather than binary scale, using a linear mixed model to examine the association between air pollutants and the continuous scale of CES-D 10. Sixth, we also performed a generalized linear mixed model with a binomial distribution and logit link similar to the GEE model specification, along with the random intercept of each subject. In addition, to examine whether the magnitude of the association between particulate matter and depression varied by subgroup, we performed a subgroup analysis by stratifying sex, age group, and population density levels. We also obtained p-values for interactions from models that included interaction terms for each subgroup and air pollution to identify statistical differences in associations. We reported our results with the odds ratio (OR) per 10 μg/m^3^ increment of concentration level and 95% confidence interval (95% CI). We also fitted a generalized additive model with the continuous score of CES-D 10 as the dependent variable and PM_2.5_ and PM_10_ as independent variables to examine any non-linear associations through spline curves.

All analyses were conducted using SAS version 9.4 (SAS Institute Inc.) and R software, version 4.1.0 (R Project for Statistical Computing).

## Results

### Descriptive information of the study population

This study included a total of 7674 participants living in 217 districts in South Korea. Descriptive information on the study participants is displayed with proportion, mean, and standard deviation by baseline year (i.e., year of the participants’ initial entries between 2016 and 2020) in Table 1. The participants were on average 68.13 years old at baseline year, and more than 57% of them were female. Most participants were non-smokers and had high school education attainment or below. Approximately 66% of the participants were non-drinkers, and 76% of the participants were married and living with spouse. Over 60% of the participants had social contact once a week or more often, 43% answered their self-reported health status as good, and only about 35% of them exercised or had private medical insurance. Most of the participants, over 87%, have resided in urban areas with at least a mid-population density. The proportions of participants who have the CES-D 10 score of 20 points or over were 22.3%, 26.3%, and 20.8% for each cohort year of 2016, 2018, and 2020, respectively.

**Table 1:** Descriptive statistics of the study population in KLoSA, 2016–2020

	Mean (SD) or n (%)
**Total study participants**	(*n* = 7674)
**Age (years)**	68.1±10.3
53–64	3230 (42.1)
65–74	2156 (28.1)
75–84	1761 (23.0)
> 85	527 (6.9)
**Sex**	
Male	3274 (42.7)
Female	4400 (57.3)
**Current smoking**	
No	6826 (89.0)
Yes	848 (11.0)
**Current drinking**	
No	5060 (65.9)
Yes	2614 (34.1)
**Education attainment**	
Primary school and below	3039 (39.6)
Middle and high school	3647 (47.5)
College and above	988 (12.9)
**Marital status**	
Single, divorced, and widowed, Married and not living with spouse	1857 (24.2)
Married and living with spouse	5817 (75.8)
**Social contact**	
Every other month or less often	1196 (15.6)
Once a month or more often and less than once a week	1736 (22.6)
Once a week or more often	4742 (61.8)
**Self-reported health status**	
Normal or bad	4394 (57.2)
Good	3280 (42.7)
**Exercise**	
No	4985 (65.0)
Yes	2689 (35.0)
**Private Medical insurance**	
No medical insurance	4915 (64.1)
Having medical insurance	2759 (36.0)
**Population density**	
Low (< 161/km^2^)	976 (12.7)
Mid (161–3591/km^2^)	2995 (39.0)
High (≥ 3591/km^2^)	3703 (48.3)
**Number of beds in hospitals per 1,000 persons**	15.0 ± 9.5
**Number of national basic livelihood beneficiaries**	7341.5 ± 5899.0
**Independent rate of finance of local government**	27.2 ± 13.5
**Proportion of basic pension beneficiaries**	69.2 ± 11.1

*KLoSA* Korean Longitudinal Study of Aging, *SD* standard deviation

### Descriptive information on air pollution

**Fig. 1:**
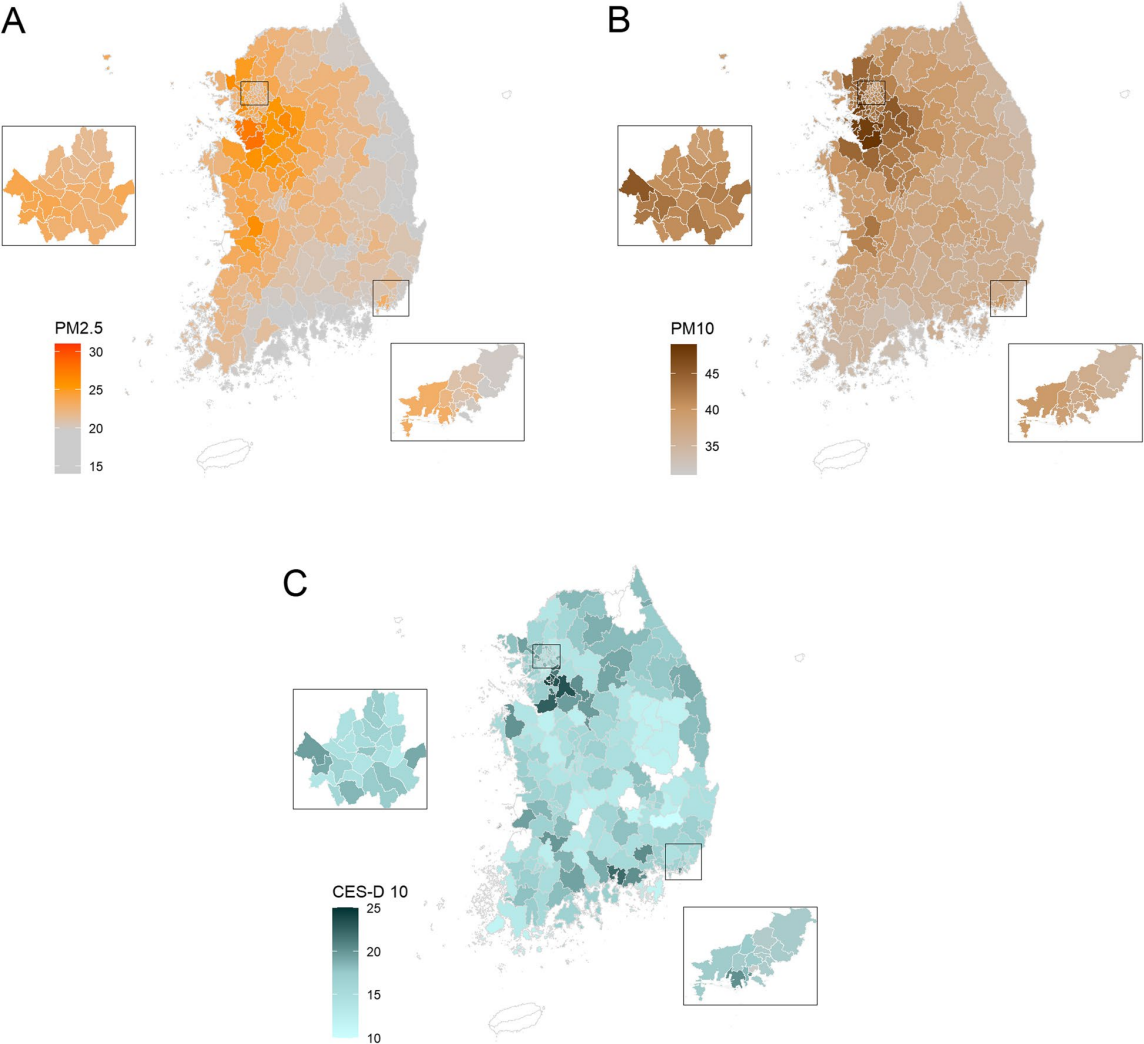
Geographical distribution of PM_2.5_, PM_10_ concentration, and CES-D 10 score averaged of 2016–2020. The map was presented for PM_2.5_ (**A**), PM_10_ (**B**), and CES-D 10 score (**C**) in South Korea. PM_2.5_: particulate matter with an aerodynamic diameter ≤ 2.5 μm; PM_10_: particulate matter with an aerodynamic diameter ≤ 10 μm; CES-D: Center for Epidemiology Studies of Depression scale

Table 2 shows the descriptive analysis results of PM_2.5_, PM_10_, and CES-D 10 score by the year of 2016, 2018, and 2020. The annual mean concentrations of PM_2.5_ were 25.48 (Standard Deviation [SD]: 2.16), 22.39 (SD: 1.65), and 18.19 (SD: 2.28) and PM_10_ were 45.17 (SD: 3.94), 39.67 (SD: 2.70), and 31.96 (SD: 3.95) in 2016, 2018, and 2020, respectively, showing the decreased trend over year. Figure 1 displays geographical distributions of the PM_2.5_ and PM_10_ concentration, and CES-D 10 score averaged of 2016–2020 in South Korea. The capital city and nearby cities (i.e., areas in the northwest) generally had higher concentration levels of PM_2.5_ and PM_10_, along with a higher CES-D 10 score, compared to other areas. The descriptive summary of the mean concentration of air pollution (PM_2.5_, PM_10_, NO_2_, and O_3_) between 2016 and 2020 by each district and the yearly concentration levels of NO_2_ and O_3_ in 2016, 2018, and 2020 are presented in the supplementary material (please see the Additional file 1: Appendix, Table A.1 and A.2).

**Table 2:** Descriptive summary of particulate matter concentrations and CES-D 10 score by KLoSA panel year

	Mean	SD	Min	Max	IQR^a^
2016					
PM_2.5_	25.48	2.16	20.65	32.62	2.93
PM_10_	45.17	3.94	36.28	56.21	5.61
CES-D 10	16.20	5.25	10.00	40.00	7.00
2018					
PM_2.5_	22.39	1.65	17.86	27.72	2.01
PM_10_	39.67	2.70	33.22	49.32	3.73
CES-D 10	16.42	5.46	10.00	39.00	8.00
2020					
PM_2.5_	18.19	2.28	13.43	23.61	3.39
PM_10_	31.96	3.95	25.25	42.65	6.31
CES-D 10	15.74	5.05	10.00	40.00	7.00

^a^Interquartile range derived by Q3-Q1

*CES-D* Center for Epidemiology Studies of Depression scale, *KLoSA* Korean Longitudinal Study of Aging, *PM*_*2.5*_ particulate matter with an aerodynamic diameter ≤ 2.5 μm, *PM*_*10*_ particulate matter with an aerodynamic diameter ≤ 10 μm, *SD* standard deviation

### Associations between long-term exposure to particulate matters and depression

Table 3 shows the associations between long-term PM_2.5_ or PM_10_ exposure and depression. In the crude GEE model estimates, the odds ratios (95% CI) for depression with a 10 $\mathrm{\mu g}/{m}^{3}$ increase were 1.28 (1.13–1.44) in PM_2.5_ and 1.15 (1.07–1.22) in PM_10_. For the main models (i.e., models additionally adjusted for individual- and regional-level characteristics from the crude models), associations were identified for both PM_2.5_ (OR: 1.36 [1.20–1.56]) and PM_10_ (OR: 1.19 [1.10–1.29]). The associations were consistently observed even after adjusting for other pollutants such as NO_2_ and O_3_ (i.e., two-pollutant models), or shifting the exposure window for one-year concentrations before the actual panel survey date instead of using the calendar-year basis annual concentrations. In addition, results from the linear mixed model showed an association between an increment in PM_2.5_ and PM_10_ and increasing CES-D 10 scores (Additional file 1: Table A.3), and consistent results in the odds ratios were identified even after applying the generalized linear mixed model (Additional file 1: Table A.4). Meanwhile, we identified generally a linear association of particulate matters on depression, within ranges between the 10th percentile and 90th percentile of each concentration distribution, through the smooth spline plots from the generalized additive model (Additional file 1: Figure A.3).

**Table 3:** Associations between long-term air pollution exposure (per 10 $\mathrm{\mu g}/{m}^{3}$ increment) and depression

	Odds Ratio (95% CI)	
	PM_2.5_	PM_10_
Crude model	1.28 (1.13, 1.44)	1.15 (1.07, 1.22)
Adjusted model^a^		
Single pollutant (main analysis)	1.36 (1.20, 1.56)	1.19 (1.10, 1.29)
Two-pollutant (plus NO_2_)	1.41 (1.22, 1.63)	1.18 (1.09, 1.28)
Two-pollutant (plus O_3_)	1.44 (1.25, 1.65)	1.20 (1.11, 1.29)
One-year average concentrations before panel examination period^b^	1.47 (1.26, 1.70)	1.25 (1.15, 1.37)

We estimated the odds ratios from the generalized estimating equation (GEE) model, and depression was defined as having a CES-D 10 score of 20 points or over

*CI* confidence interval, *PM*_*2.5*_ particulate matter with an aerodynamic diameter ≤ 2.5 μm, *PM*_*10*_ particulate matter with an aerodynamic diameter ≤ 10 μm, *NO*_*2*_ nitrogen dioxide, *O*_*3*_ ozone

^a^Adjusted for baseline depression status, sex, age group, current smoking, current drinking, education attainment, marital status, social contact, self-reported health status, exercise, private medical insurance, longitude, latitude, and the interaction term of longitude and latitude, population density, the number of beds in hospitals per 1,000 persons, the number of national basic livelihood beneficiaries, the independent rate of finance of local government, and the proportion of basic pension beneficiaries

^b^Average concentration from September of the previous year to August of the current year in 2016, 2018, and August of the previous year to July of the current year in 2020

### Subgroup analysis

**Fig. 2:**
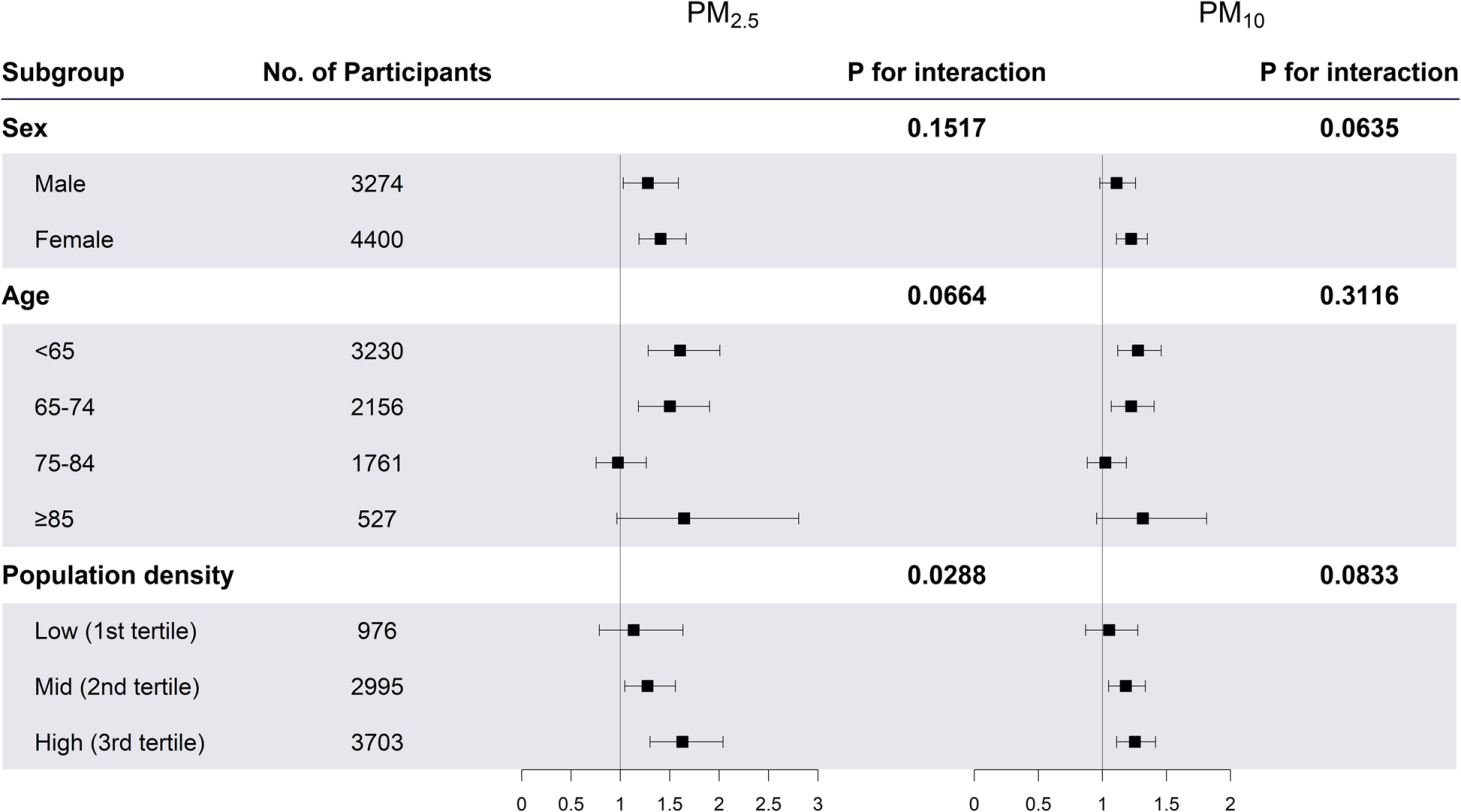
Associations between long-term air pollution exposure (per 10 $\mathrm{\mu g}/{m}^{3}$ increment) and depression. The square symbol represents odds ratio and the horizontal line represents 95% confidence interval. The odds ratio was estimated from the generalized estimating equation (GEE) model, and depression was defined as having a CES-D 10 score of 20 points or over. The models were adjusted for baseline depression status, sex, age group, current smoking, current drinking, education attainment, marital status, social contact, self-reported health status, exercise, private medical insurance, longitude, latitude, and the interaction term of longitude and latitude, population density, the number of beds in hospitals per 1,000 persons, the number of national basic livelihood beneficiaries, the independent rate of finance of local government, and the proportion of basic pension beneficiaries except for the subgroup variable itself in the model. The p-values for interactions were calculated from models that included interaction terms for each subgroup and air pollution. PM_2.5_: particulate matter with an aerodynamic diameter ≤ 2.5 μm; PM_10_: particulate matter with an aerodynamic diameter ≤ 10 μm

Figure 2 depicts the associations between long-term PM_2.5_ or PM_10_ exposure per 10 $\mathrm{\mu g}/{m}^{3}$ increment and depression by the subgroups of sex, age group, and population density. For the sex subgroup, the female group (OR [95% CI]; PM_2.5_: 1.41 [1.19–1.67], PM_10_: 1.22 [1.11–1.35]) had larger associations than the male group (PM_2.5_: 1.28 [1.03–1.59], PM_10_: 1.11 [0.98–1.26]), although the interactions were not statistically significant. No significant difference in the magnitude of association was found for the age subgroup. For the population density subgroup, we found that urban regions including high-population-density areas (PM_2.5_: 1.63 [1.30–2.04], PM_10_: 1.25 [1.11–1.42]) and mid-population-density areas (PM_2.5_: 1.27 [1.05–1.56], PM_10_: 1.18 [1.05–1.33]) had prominent associations for both PM_2.5_ and PM_10_ with depression, in contrast to the low-population density areas, showing no significant association.

## Discussion

This study examined the association between particulate matters including PM_2.5_ and PM_10_ and depression in the elderly population, using the depression index of the CES-D in the panel-surveyed longitudinal data of 2016, 2018, and 2020 in South Korea, as well as the annual concentrations of particulate matters derived from the machine learning-based ensemble models with high spatial resolution and performance, which enabled us to cover the majority areas of the country. We found that higher levels of concentration of both PM_2.5_ and PM_10_ were associated with an increased risk of depression in the elderly, with slightly higher risks in PM_2.5_ than in PM_10_. In addition, the impacts of particulate matters on depression were greater in the female group compared to the male group, although the difference in magnitude of association was marginally significant only in PM_10_. We also found that associations were only observed in urban areas with higher population densities, whereas no significant association was identified in low-population-density areas.

The results of this study are consistent with findings from previous studies that long-term exposures to PM_2.5_ and PM_10_ are associated with an increased risk of depression in adults [15, 18–24, 27, 28]. A longitudinal study conducted in the capital city of South Korea with participants aged 15–79 years (*n* = 27,270) reported that an increased risk of a diagnosis of major depressive disorder, which was defined by the ICD-10 code F32, was observed (hazard ratio [HR]: 1.47, 95% CI: 1.14–1.90) with a 10 $\mathrm{\mu g}/{m}^{3}$ increase in the 12-month moving average concentration of PM_2.5_ [19]. Another cohort study in South Korea (*n* = 123,045) showed that an increase of depression, defined by the ICD-10 code F31-33, was associated with a 10 $\mathrm{\mu g}/{m}^{3}$ increase in the 12-month average concentration of PM_10_ (HR: 1.11, 95% CI: 1.06–1.16) [21]. In South Korea, the impact size of the long-term association between air pollution and depression, represented as RR or HR and 95% CIs, varied from 1.01 (0.83–1.22) to 1.47 (1.14–1.90) for PM_2.5_, and from 1.03 (1.00–1.05) to 1.15 (1.05–1.28) for PM_10_ (please see the Additional file 1: Appendix, Table A.5). Meanwhile, a cohort study conducted in China (*n* = 30,712) reported that an increased risk of depression, defined by ICD-10 code F32, was associated with a 10 $\mathrm{\mu g}/{m}^{3}$ increase in the one-year average concentration of PM_2.5_ (HR: 1.88, 95% CI: 1.56–2.26) and PM_10_ (HR: 1.35, 95% CI: 1.23–1.49) in the elderly population [24]. Another longitudinal study for elderly people in the US (*n* = 8,907,422) showed an increased risk of depression (HR: 1.04, 95% CI: 1.03–1.06) associated with a 10 $\mathrm{\mu g}/{m}^{3}$ increase in the past 5-year average concentration of PM_2.5_ [27]. However, some studies could not find an association between particulate matter and depression in the elderly population [25, 26, 29]. Among the studies conducted in other regions outside of South Korea, the impact size of the long-term association between air pollution and depression, which were represented as OR or HR and 95% CIs with a 10 μg/m^3^ increase in concentration, ranged from 1.04 (1.03–1.06) to 1.88 (1.26–2.26) for PM_2.5_, and from 0.99 (0.95–1.05) to 1.35 (1.23–1.49) for PM_10_, except for one study conducted in Germany [28], which reported notably high ORs (PM_2.5_: 14.59 [1.38–148.55], PM_10_: 2.76 [0.75–3.69]) (Additional file 1: Table A.5). We conjecture that the discrepancy in the results might have resulted from the difference in participant characteristics and the diversity in regional characteristics such as urbanicity. In addition, different types of measurements defining depression, or a discrepancy in the background concentration level of air pollution may also contribute to the divergent results (i.e., a higher air pollution concentration level in Asia compared to the US or Europe) (Additional file 1: Table A.5).

There are several plausible mechanisms that can explain the negative impacts of particulate matters on depression. It has been presumed that exposure to air pollution can affect the functioning of dopaminergic neurons and neurotransmitters like serotonin [9], and PM_2.5_ appears especially harmful because it enters the circulation and causes inflammatory reactions in the respiratory tract, which then causes systemic inflammation and the release of inflammatory mediators [10, 38]. Moreover, it has been proposed that PM_2.5_ can pass the lung tissue compartment, enter the bloodstream, and travel directly to the brain, where it can alter neuronal morphology and inflammatory cytokines in the hippocampus or cause oxidative stress [39] and inflammation in the central nervous system [10, 13]. In terms of aging, the physiological changes in the immune responses have been considered remarkable in older people. As an aging-related progression, it can be explained that aging stimulates peripheral immune responses, affecting peripheral-brain immunological interaction, and leading to the production of pro-inflammatory cytokines, which may aggravate depressive symptoms [16]. Besides, other factors such as physical aging, social problems in relationships, and economic status could also affect personal mood [40], with the fact that older people are more likely to experience socio-economic disadvantages [41].

This study also found that the impacts of particulate matters on depression were slightly greater in the female group compared to the male group. Several previous studies reported that women had higher odds of depressive disorders when exposed to higher concentrations of air pollution [42, 43], explaining that elderly women are more vulnerable to social effects and poor health conditions, thus they are more likely to be depressed than men [44]. However, research conducted in the US showed that the hazard ratios in depression diagnosis risk per 5-unit increase in PM_2.5_ concentration were 1.02 (95% CI: 1.01, 1.03) in the male group and 1.01 (95% CI: 1.00, 1.02) in the female group [27]. Therefore, along with the insignificant result in the effect modification by sex subgroup in this study, these conflicting results need to be further investigated in future studies.

Interestingly, in contrast to the result observed in the high-population-density areas, which showed strong associations with depression, the low-population-density areas did not show an evident association with depression in this study. In addition, to the best of our knowledge, this is the first study that examines regional differences in the association between particulate matters and depression in the elderly in South Korea. We conjecture that the results of the rural areas are related to their residential environment: people who live in relatively low-populated regions in South Korea have more chance to live in regions with a lot of mountains and greenery, and such greenness could have a protective influence on depression [45]. In contrast, people who live in urban cities generally may have a lower level of mental health than those who live in low-populated regions, which could make them more sensitive to the environmental effects related to mental health outcomes [46].

This study had several limitations. First, the CES-D 10 depression scores used in this study were based on a self-reported questionnaire, which was not a medical diagnosis; therefore, a careful interpretation would be needed for the results, considering the potential bias such as recall bias. Nevertheless, the CES-D 10 is a widely used tool to evaluate depression, and its validity and reliability have been verified in many studies [47–49]. Second, in this study, aggregated district-level yearly air pollution concentrations were linked to individuals due to the limitation that participants’ addresses in the KLoSA were only available at the district level. Thus exposure misclassification may exist and air pollution concentrations were calculated based on outdoor concentrations, which also could not represent indoor concentrations. Nevertheless, districts in South Korea are second-level local authority areas (shi/gun/gu) within metropolitan cities and provinces, with a median area size (397 km^2^) of approximately 1.7 times that of a ZIP code (233 km^2^) in the United States. Therefore, we speculate that the impact of air pollution can be sufficiently captured in the nationwide-scale environmental epidemiologic study [34, 50, 51], despite the limitation of reflecting precise personal-level exposures. Third, our study, as a panel study, deviates from the cohort study paradigm by not involving the prospective tracking and observation of individuals without the disease at the baseline. Instead, it constitutes a repeated measurement investigation, wherein the probability of depression and the association with air pollution were observed across multiple time points.

Despite the limitations, this is the first study that investigated long-term associations of exposure to PM_2.5_ and PM_10_ on depression in the elderly population, with nationwide longitudinal data using individual socioeconomic characteristics and air pollutant prediction models that enabled us to cover most of the regions with a high spatial resolution in South Korea. In addition, this study found a piece of evidence that regional factors may have a role in the associations between air pollution and depression, from this implication, further research can investigate the roles of the regional factors in depth.

## Conclusions

In summary, we found that higher levels of annual exposure to PM_2.5_ and PM_10_ were associated with increased risks of depression in the elderly in South Korea, along with the results that associations differed by region, which was characterized by population density level. This study provides quantitative evidence of the negative impacts of particulate matters for potential patients to have depression and suggests beneficial implications for policymakers to establish public health policies regarding air pollution and mitigate the socioeconomic burden contributed by depression in the elderly population.

### Supplementary Information

**Additional file 1:** Study Population. **Figure A.1.** Flowchart of participant eligibility in the current study. Air Pollution Prediction Model. **Figure A.2.** Flow diagram of the air pollution prediction modeling process. **Supplementary Results**. **Table A.1.** Average concentrations of air pollution during the study period (2016, 2018, 2020) and regional characteristics of 217 districts in South Korea. **Table A.2.** Descriptive summary of air pollution concentrations (NO_2_ and O_3_). **Table A.3.** Associations between long-term air pollution exposure (per 10 μg/m^3^ increment) and changes in CES-D 10 score by linear mixed models. **Table A.4.** Associations between long-term air pollution exposure (per 10 μg/m^3^ increment) and depression by generalized linear mixed models. **Table A.5.** Summary of the previous studies on the long-term association between particulate matters and depression. **Figure A.3.** Exposure-response curves of the particulate matters (PM_2.5_ and PM_10_) on depression based on the continuous scale of the CES-D 10 score.

## Data Availability

The KLoSA (Korean longitudinal study of aging) data is publicly available for the basic version, which provides participants' addresses at the province level. However, a data request and permission process are needed by the Korea Employment Information Service (KEIS) to obtain the data with participants' addresses at the district level that this study used. The air pollution prediction data provided by the AiMS-CREATE network is publicly available when there is no conflict in study topics, through a brief data request on the website (URL: https://www.datascience4health.com/).

## Abbreviations

KLoSA: Korean longitudinal study of aging
CES-D: Center for epidemiology studies of depression scale
PM: Particulate matter
PM_2.5_: Particulate matter with diameter less than 2.5 μm
PM_10_: Particulate matter with diameter less than 10 $\mu m$
NO_2_: Nitrogen dioxide
O_3_: Ozone
OR: Odds ratio
CI: Confidence interval
