# Supplementary material for: Particulate matters (PM2.5, PM10) and the risk of depression among middle-aged and older population: analysis of the Korean Longitudinal Study of Aging (KLoSA), 2016–2020 in South Korea
Source: Environ Health. 2024 Jan 3;23:4. doi: 10.1186/s12940-023-01043-1 (PMC10762940; doi:10.1186/s12940-023-01043-1)
Supplement: Supplementary file 1 — Additional file 1: Study Population. Figure A.1. Flowchart of participant eligibility in the current study. Air Pollution Prediction Model. Figure A.2. Flow diagram of the air pollution prediction modeling process. Supplementary Results. Table A.1. Average concentrations of air pollution during the study period (2016, 2018, 2020) and regional characteristics of 217 districts in South Korea. Table A.2. Descriptive summary of air pollution concentrations (NO2 and O3). Table A.3. Associations between long-term air pollution exposure (per 10 μg/m3 increment) and changes in CES-D 10 score by linear mixed models. Table A.4. Associations between long-term air pollution exposure (per 10 μg/m3 increment) and depression by generalized linear mixed models. Table A.5. Summary of the previous studies on the long-term association between particulate matters and depression. Figure A.3. Exposure-response curves of the particulate matters (PM2.5 and PM10) on depression based on the continuous scale of the CES-D 10 score. [file 12940_2023_1043_MOESM1_ESM.docx]

# **Supplementary data**

**Title:** Particulate Matters (PM_2.5_, PM_10_) and the risk of depression among middle-aged and older population: analysis of the Korean Longitudinal Study of Aging (KLoSA), 2016-2020 in South Korea.

**Authors**

Hyunkyung Park, Cinoo Kang, AiMS-CREATE Team, and Ho Kim*

HP and CK contributed equally to this article as co-first authors.

**Table of Contents**

1. **Study Population**

**Figure A.1.** Flowchart of participant eligibility in the current study.

1. **Air Pollution Prediction Model**

**Figure A.2.** Flow diagram of the air pollution prediction modeling process.

1. **Supplementary Results**

**Table A.1.** Average concentrations of air pollution during the study period (2016, 2018, 2020) and regional characteristics of 217 districts in South Korea.

**Table A.2.** Descriptive summary of air pollution concentrations (NO_2_ and O_3_).

**Table A.3.** Associations between long-term air pollution exposure (per 10 μg/m^3^ increment) and changes in CES-D 10 score by linear mixed models.

**Table A.4.** Associations between long-term air pollution exposure (per 10 μg/m^3^ increment) and depression by generalized linear mixed models.

**Table A.5.** Summary of the previous studies on the long-term association between particulate matters and depression.

**Figure A.3.** Exposure-response curves of the particulate matters (PM_2.5_ and PM_10_) on depression based on the continuous scale of the CES-D 10 score.

**1. Study Population**

**Figure A.1. Flowchart of participant eligibility in the current study.**


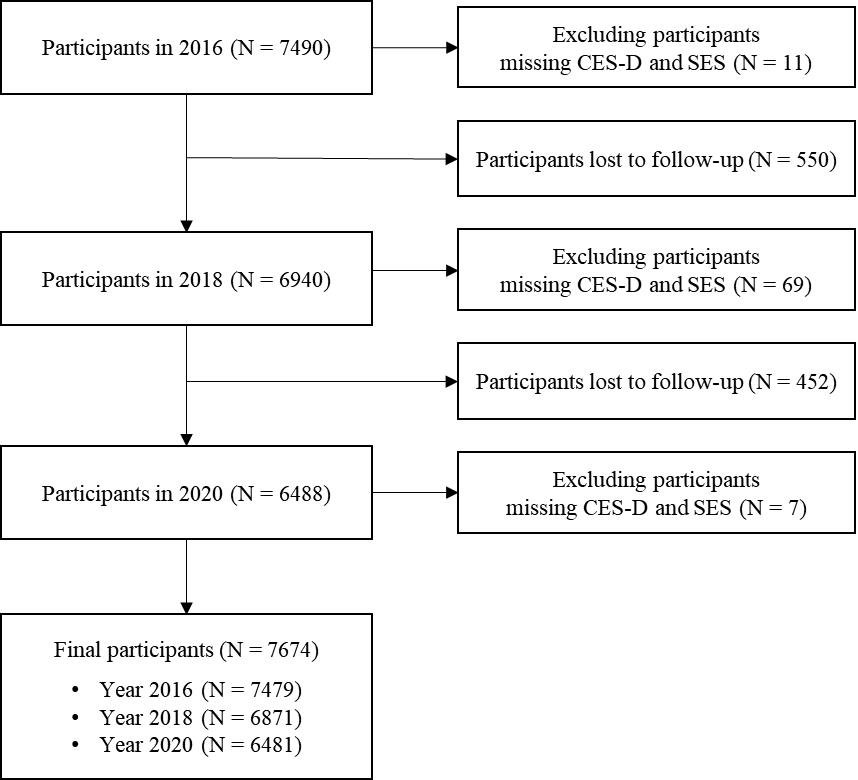


**2. Air Pollution Prediction Model**

Air pollution prediction models were developed by AiMS-CREATE network. For the full process and result of prediction model and more details can be found below:
<https://papers.ssrn.com/sol3/papers.cfm?abstract_id=4280944>

*1.1 Modeling procedures*

To establish the prediction model, predictor variables were collected from the google earth engine (GEE), Socioeconomic Data and Applications Center (SEDAC), and regional socioeconomic database as input variables, and each of the five air pollution concentrations (PM_2.5_, PM_10_, NO_2_, O_3_, and CO) were predicted as the outcome variables. Missing values in the predictor variables were replaced with values through the imputation process. Randomly selected 80% of the monitoring stations were used to train the models, and the remaining 20% of the monitoring stations were used to test the performance of the model. Each of the three machine learning models (i.e., random forest, light gradient boosting, and neural network model) was trained with 10-fold cross-validation (CV) in the training set to avoid overfitting problems. We validated the model performance using R^2^ and root mean squared error (RMSE) with three machine learning models and simple averages among models (ensemble model) in the test set. Finally, we predicted monthly basis air pollution concentrations with a 1 km × 1 km grid during 2002–2020 using the three machine learning-based models and ensemble models.

*1.2 Ensemble model*

To aggregate the predictions, we calculated the simple averages of each machine learning estimation.

$$\hat{Y}_{SAij}= \frac{\hat{Y}_{RFij}+ \hat{Y}_{GBij}+ \hat{Y}_{NNij}}{3}$$

$\hat{Y}_{RFij}$, $\hat{Y}_{GBij}$, 𝑎𝑛𝑑 $\hat{Y}_{NNij}$ are predicted air pollution concentrations from the random forest, light gradient boosting, and neural network, respectively, at location i at time j. 𝑌_𝑆𝐴𝑖𝑗_ is the simple averaged prediction values derived by averaging the three estimations at location i at time j. We also trained a generalized additive model (GAM) to consider the geographical variation of each of the three machine-learning estimations.

**Figure A.2. Flow diagram of the air pollution prediction modeling process.** CV, cross-validation; RF, random forest; GB, light gradient boosting; NN, neural network model.


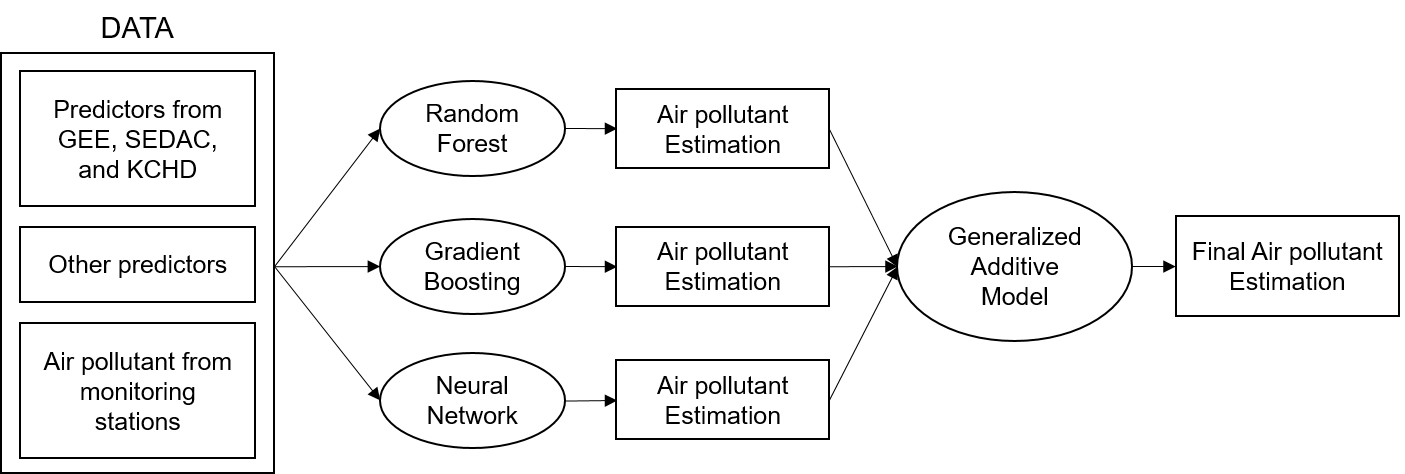


[Overall process]

**
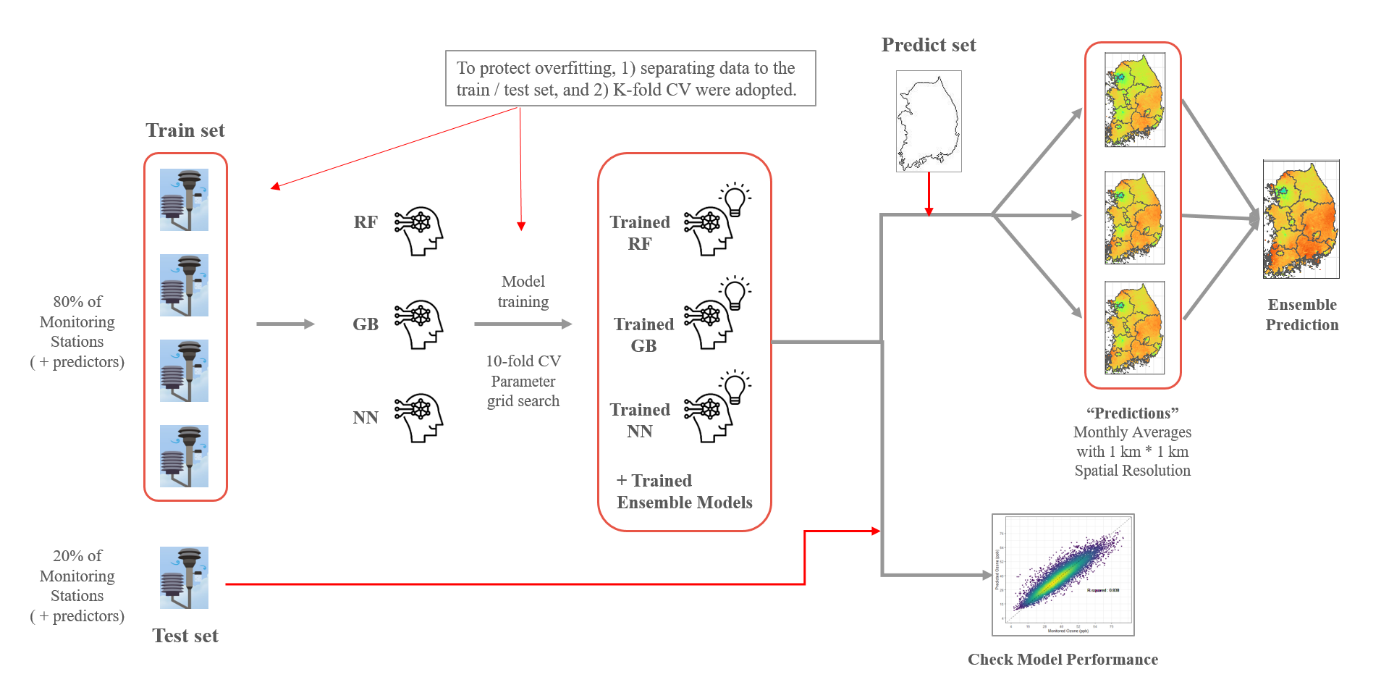
**

[Train, validation, and prediction process]

**2. Supplementary Results**

**Table A.1 Average concentrations of air pollution during the study period (2016, 2018, 2020) and regional characteristics of 217 districts in South Korea.**

| No | Province | District | PM_2.5_ | PM_10_ | NO_2_ | O_3_ | Longitude | Latitude | Population  density | No. of beds in hospitals per 1,000 persons | Independent rate of finance of local government | No. of national basic livelihood beneficiaries | % of basic pension beneficiaries |
| --- | --- | --- | --- | --- | --- | --- | --- | --- | --- | --- | --- | --- | --- |
| 1 | Seoul | Jongno-gu | 22.04 | 40.74 | 24.04 | 38.11 | 126.98 | 37.59 | 6680.47 | 20.74 | 53.63 | 4496.33 | 46.45 |
| 2 | Seoul | Jung-gu | 22.70 | 39.92 | 27.01 | 35.54 | 127.00 | 37.56 | 12985.55 | 11.22 | 64.83 | 4283.00 | 52.36 |
| 3 | Seoul | Yongsan-gu | 23.01 | 40.91 | 25.55 | 36.68 | 126.98 | 37.53 | 10891.20 | 3.87 | 46.60 | 6483.33 | 40.83 |
| 4 | Seoul | Eunpyung-gu | 22.06 | 40.25 | 23.43 | 39.13 | 126.93 | 37.62 | 16760.45 | 5.57 | 23.47 | 19369.67 | 63.24 |
| 5 | Seoul | Seongdong-gu | 22.89 | 42.76 | 26.98 | 34.75 | 127.04 | 37.55 | 17720.08 | 5.37 | 39.13 | 7849.33 | 50.69 |
| 6 | Seoul | Gwangjin-gu | 23.17 | 42.48 | 26.30 | 35.26 | 127.09 | 37.54 | 21421.67 | 5.91 | 32.87 | 8664.00 | 48.48 |
| 7 | Seoul | Dongdaemun-gu | 22.77 | 41.05 | 27.20 | 34.96 | 127.06 | 37.58 | 25475.77 | 12.96 | 28.00 | 12421.33 | 57.86 |
| 8 | Seoul | Jungnang-gu | 22.47 | 41.16 | 25.46 | 36.60 | 127.10 | 37.60 | 22558.82 | 5.82 | 23.93 | 17383.00 | 65.63 |
| 9 | Seoul | Seongbuk-gu | 22.06 | 41.58 | 25.59 | 36.68 | 127.02 | 37.60 | 19124.70 | 5.86 | 26.10 | 13172.00 | 58.51 |
| 10 | Seoul | Nowon-gu | 21.90 | 40.24 | 23.75 | 39.20 | 127.08 | 37.65 | 16538.18 | 5.38 | 20.30 | 26264.00 | 66.86 |
| 11 | Seoul | Gangbuk-gu | 21.67 | 38.91 | 23.71 | 38.69 | 127.01 | 37.64 | 14267.60 | 5.11 | 24.33 | 16407.67 | 69.17 |
| 12 | Seoul | Gangseo-gu | 24.06 | 46.01 | 24.62 | 37.84 | 126.83 | 37.56 | 13980.47 | 4.81 | 26.77 | 22491.67 | 61.02 |
| 13 | Seoul | Dobong-gu | 21.73 | 39.23 | 22.73 | 40.14 | 127.04 | 37.67 | 17228.85 | 6.04 | 24.90 | 11331.00 | 64.46 |
| 14 | Seoul | Seodaemun-gu | 22.58 | 40.66 | 25.35 | 37.31 | 126.94 | 37.58 | 17803.75 | 10.04 | 31.53 | 8535.67 | 58.38 |
| 15 | Seoul | Mapo-gu | 23.07 | 41.13 | 25.38 | 36.61 | 126.91 | 37.56 | 16142.07 | 2.13 | 39.97 | 8271.33 | 51.13 |
| 16 | Seoul | Yangcheon-gu | 23.95 | 42.93 | 26.99 | 36.18 | 126.86 | 37.52 | 28127.75 | 4.90 | 30.17 | 12196.33 | 58.04 |
| 17 | Seoul | Geumcheon-gu | 23.36 | 40.74 | 26.41 | 37.24 | 126.90 | 37.46 | 18416.32 | 6.75 | 29.87 | 9963.00 | 60.90 |
| 18 | Seoul | Guro-gu | 23.79 | 43.03 | 26.43 | 36.45 | 126.86 | 37.49 | 21086.00 | 7.96 | 27.13 | 9300.00 | 57.63 |
| 19 | Seoul | Yeongdeungpo-gu | 23.64 | 42.99 | 25.41 | 36.29 | 126.91 | 37.52 | 15667.05 | 11.85 | 43.50 | 8635.00 | 45.43 |
| 20 | Seoul | Dongjak-gu | 23.34 | 40.98 | 26.75 | 35.93 | 126.95 | 37.50 | 24733.05 | 5.88 | 32.13 | 9306.67 | 48.71 |
| 21 | Seoul | Gangnam-gu | 22.69 | 41.08 | 26.51 | 36.07 | 127.07 | 37.49 | 14409.32 | 13.61 | 66.37 | 11975.00 | 28.13 |
| 22 | Seoul | Gwanak-gu | 22.84 | 41.15 | 25.00 | 37.80 | 126.95 | 37.46 | 17457.33 | 3.76 | 25.57 | 14581.00 | 55.93 |
| 23 | Seoul | Seocho-gu | 22.86 | 42.76 | 24.38 | 37.90 | 127.03 | 37.47 | 9411.62 | 7.36 | 63.23 | 5446.33 | 25.95 |
| 24 | Seoul | Songpa-gu | 22.97 | 41.38 | 26.91 | 36.56 | 127.12 | 37.50 | 19712.22 | 8.16 | 47.83 | 11669.33 | 37.90 |
| 25 | Seoul | Gangdong-gu | 23.78 | 43.09 | 25.71 | 36.22 | 127.15 | 37.55 | 19290.93 | 10.37 | 35.90 | 10715.33 | 51.36 |
| 26 | Busan | Jung-gu | 23.11 | 38.59 | 21.86 | 37.74 | 129.03 | 35.10 | 16636.77 | 27.31 | 30.10 | 3038.67 | 70.68 |
| 27 | Busan | Seo-gu | 21.44 | 37.25 | 18.48 | 40.43 | 129.02 | 35.10 | 8505.38 | 35.96 | 16.73 | 7857.33 | 74.12 |
| 28 | Busan | Dong-gu | 20.79 | 37.29 | 20.54 | 38.86 | 129.05 | 35.13 | 9783.98 | 24.17 | 17.97 | 7722.67 | 77.97 |
| 29 | Busan | Nam-gu | 20.26 | 37.66 | 19.73 | 40.16 | 129.10 | 35.12 | 10736.28 | 8.82 | 31.77 | 9398.67 | 71.15 |
| 30 | Busan | Yeongdo-gu | 19.73 | 36.86 | 17.98 | 41.34 | 129.07 | 35.08 | 9504.48 | 9.76 | 13.70 | 10578.33 | 76.00 |
| 31 | Busan | Busanjin-gu | 20.94 | 36.87 | 20.19 | 39.70 | 129.05 | 35.16 | 13040.82 | 14.16 | 24.80 | 15867.00 | 71.91 |
| 32 | Busan | Sasang-gu | 22.71 | 39.73 | 19.41 | 39.52 | 128.99 | 35.15 | 6766.97 | 19.96 | 24.00 | 12350.33 | 74.44 |
| 33 | Busan | Dongrae-gu | 21.94 | 38.47 | 20.35 | 40.27 | 129.08 | 35.20 | 16640.98 | 15.99 | 23.10 | 8404.67 | 65.56 |
| 34 | Busan | Gangseo-gu | 22.48 | 38.73 | 16.20 | 41.67 | 128.91 | 35.15 | 444.25 | 2.44 | 49.93 | 2848.33 | 59.74 |
| 35 | Busan | Haeundae-gu | 20.19 | 35.82 | 18.40 | 41.75 | 129.16 | 35.19 | 8243.47 | 11.04 | 30.53 | 16508.33 | 64.08 |
| 36 | Busan | Buk-gu | 21.04 | 36.21 | 17.91 | 41.72 | 129.03 | 35.23 | 7868.27 | 11.05 | 16.33 | 17226.33 | 71.51 |
| 37 | Busan | Saha-gu | 23.36 | 39.02 | 19.07 | 39.93 | 128.98 | 35.09 | 8278.42 | 15.57 | 20.30 | 16619.33 | 73.42 |
| 38 | Busan | Geumjeong-gu | 20.97 | 37.02 | 17.07 | 42.94 | 129.09 | 35.26 | 3843.05 | 16.80 | 23.83 | 10434.67 | 67.10 |
| 39 | Busan | Yeonje-gu | 21.65 | 38.82 | 21.04 | 39.88 | 129.09 | 35.18 | 17378.40 | 16.87 | 23.67 | 8147.67 | 67.48 |
| 40 | Busan | Suyeong-gu | 21.96 | 37.78 | 19.98 | 40.14 | 129.11 | 35.16 | 17410.45 | 16.41 | 24.40 | 6327.67 | 63.57 |
| 41 | Busan | Gijang-gun | 20.00 | 34.67 | 14.12 | 43.98 | 129.20 | 35.30 | 619.68 | 8.55 | 38.17 | 7002.67 | 71.18 |
| 42 | Daegu | Jung-gu | 21.32 | 39.67 | 22.31 | 38.63 | 128.60 | 35.86 | 11049.92 | 39.29 | 31.17 | 4093.33 | 66.70 |
| 43 | Daegu | Buk-gu | 21.44 | 39.47 | 18.34 | 41.52 | 128.58 | 35.93 | 4731.05 | 10.46 | 21.53 | 15680.33 | 72.16 |
| 44 | Daegu | Dalseo-gu | 20.95 | 37.71 | 19.31 | 40.50 | 128.53 | 35.83 | 9689.77 | 10.21 | 25.63 | 25057.67 | 67.17 |
| 45 | Daegu | Dong-gu | 21.18 | 38.00 | 16.31 | 42.58 | 128.69 | 35.93 | 1899.33 | 8.06 | 19.50 | 16957.33 | 71.02 |
| 46 | Daegu | Seo-gu | 23.11 | 41.66 | 21.47 | 38.73 | 128.55 | 35.87 | 12218.63 | 14.14 | 18.23 | 11820.33 | 78.69 |
| 47 | Daegu | Nam-gu | 21.06 | 37.80 | 20.02 | 40.54 | 128.59 | 35.83 | 9412.47 | 23.65 | 15.53 | 11923.00 | 71.85 |
| 48 | Daegu | Suseong-gu | 20.75 | 37.41 | 18.39 | 41.42 | 128.66 | 35.83 | 5966.02 | 6.58 | 29.40 | 17839.67 | 56.09 |
| 49 | Daegu | Dalseong-gun | 20.05 | 37.29 | 13.98 | 43.61 | 128.51 | 35.73 | 447.30 | 7.70 | 41.00 | 8579.00 | 69.26 |
| 50 | Incheon | Bupyeong-gu | 23.05 | 42.25 | 25.36 | 36.61 | 126.72 | 37.49 | 17392.55 | 7.51 | 23.03 | 24183.33 | 70.96 |
| 51 | Incheon | Jung-gu | 22.04 | 39.02 | 17.67 | 41.80 | 126.47 | 37.47 | 808.80 | 12.67 | 51.43 | 4689.00 | 66.91 |
| 52 | Incheon | Dong-gu | 23.20 | 45.03 | 26.49 | 35.03 | 126.64 | 37.48 | 10386.03 | 8.58 | 30.13 | 3147.00 | 77.86 |
| 53 | Incheon | Yeonsu-gu | 22.05 | 41.73 | 22.18 | 39.78 | 126.65 | 37.37 | 6335.65 | 4.08 | 44.60 | 10032.33 | 62.16 |
| 54 | Incheon | Namdong-gu | 23.14 | 44.01 | 24.69 | 37.76 | 126.73 | 37.43 | 9010.90 | 9.68 | 32.50 | 21570.33 | 70.87 |
| 55 | Incheon | Gyeyang-gu | 24.20 | 43.71 | 21.96 | 38.74 | 126.74 | 37.55 | 7440.58 | 9.37 | 23.57 | 11380.00 | 76.33 |
| 56 | Incheon | Seo-gu | 23.03 | 43.20 | 21.24 | 39.32 | 126.65 | 37.56 | 4221.40 | 7.65 | 40.33 | 15445.00 | 71.90 |
| 57 | Incheon | Ganghwa-gun | 22.67 | 38.52 | 12.86 | 43.61 | 126.45 | 37.70 | 163.30 | 9.56 | 17.27 | 3454.00 | 65.18 |
| 58 | Incheon | Nam-gu | 23.47 | 43.78 | 25.96 | 35.93 | 126.67 | 37.45 | 16600.60 | 10.46 | 20.77 | 16174.00 | 72.41 |
| 59 | Gwangju | Dong-gu | 20.70 | 37.07 | 16.07 | 42.82 | 126.95 | 35.11 | 2067.15 | 45.00 | 16.33 | 6744.33 | 62.03 |
| 60 | Gwangju | Seo-gu | 20.56 | 37.41 | 18.03 | 41.42 | 126.85 | 35.13 | 6539.07 | 16.17 | 23.67 | 14374.00 | 61.25 |
| 61 | Gwangju | Buk-gu | 20.74 | 36.80 | 16.50 | 42.85 | 126.93 | 35.19 | 3697.30 | 17.49 | 15.83 | 24800.67 | 67.79 |
| 62 | Gwangju | Nam-gu | 21.92 | 37.65 | 16.32 | 42.62 | 126.86 | 35.09 | 3591.65 | 20.79 | 13.43 | 10808.33 | 66.45 |
| 63 | Gwangju | Gwangsan-gu | 21.59 | 35.97 | 14.69 | 44.01 | 126.76 | 35.16 | 1760.65 | 16.61 | 21.90 | 15880.33 | 72.10 |
| 64 | Daejeon | Dong-gu | 21.44 | 38.49 | 16.35 | 43.00 | 127.48 | 36.32 | 1797.67 | 9.80 | 13.03 | 14178.67 | 73.57 |
| 65 | Daejeon | Seo-gu | 20.51 | 38.18 | 16.82 | 42.33 | 127.35 | 36.28 | 5188.62 | 11.91 | 22.53 | 15083.00 | 60.38 |
| 66 | Daejeon | Jung-gu | 20.56 | 38.53 | 16.77 | 42.74 | 127.41 | 36.28 | 4196.15 | 17.54 | 16.17 | 11919.67 | 66.17 |
| 67 | Daejeon | Yuseong-gu | 21.26 | 39.06 | 15.76 | 42.92 | 127.34 | 36.37 | 2072.82 | 8.95 | 33.70 | 6448.00 | 53.50 |
| 68 | Daejeon | Daedeok-gu | 23.35 | 42.67 | 17.83 | 42.03 | 127.44 | 36.41 | 2929.30 | 10.49 | 20.97 | 9273.00 | 71.32 |
| 69 | Ulsan | Jung-gu | 20.78 | 37.73 | 18.42 | 40.88 | 129.31 | 35.57 | 6423.30 | 7.83 | 20.77 | 5564.67 | 65.53 |
| 70 | Ulsan | Buk-gu | 20.17 | 36.56 | 15.97 | 42.44 | 129.38 | 35.61 | 1194.23 | 4.85 | 32.63 | 3223.00 | 66.24 |
| 71 | Ulsan | Nam-gu | 20.86 | 38.02 | 20.01 | 39.67 | 129.33 | 35.51 | 4738.75 | 13.59 | 37.97 | 6206.00 | 58.53 |
| 72 | Ulsan | Dong-gu | 19.33 | 36.95 | 17.90 | 40.43 | 129.43 | 35.52 | 4867.43 | 9.41 | 26.03 | 3156.00 | 62.11 |
| 73 | Ulsan | Ulju-gun | 20.28 | 36.83 | 13.86 | 43.86 | 129.19 | 35.54 | 280.45 | 10.41 | 45.20 | 4570.33 | 66.19 |
| 74 | Gyeonggi | Ansan-si | 23.95 | 44.34 | 21.35 | 40.73 | 126.84 | 37.32 | 4697.68 | 10.27 | 53.93 | 22461.67 | 65.31 |
| 75 | Gyeonggi | Uijeongbu-si | 22.89 | 43.02 | 20.08 | 41.62 | 127.07 | 37.73 | 5302.12 | 9.03 | 31.60 | 16191.67 | 68.69 |
| 76 | Gyeonggi | Goyang-si | 24.60 | 44.91 | 20.50 | 40.55 | 126.80 | 37.67 | 3728.70 | 9.01 | 48.23 | 23231.33 | 59.00 |
| 77 | Gyeonggi | Yangju-si | 23.61 | 42.88 | 15.50 | 43.70 | 127.00 | 37.81 | 651.25 | 8.43 | 35.57 | 7333.33 | 68.29 |
| 78 | Gyeonggi | Guri-si | 24.15 | 44.64 | 22.44 | 39.13 | 127.13 | 37.60 | 5725.00 | 9.89 | 38.17 | 6011.67 | 61.64 |
| 79 | Gyeonggi | Anyang-si | 24.05 | 44.71 | 24.37 | 38.92 | 126.94 | 37.40 | 10349.80 | 6.24 | 50.50 | 10436.67 | 58.50 |
| 80 | Gyeonggi | Gimpo-si | 26.64 | 45.30 | 17.66 | 41.94 | 126.63 | 37.68 | 1151.10 | 6.19 | 47.43 | 7853.67 | 58.42 |
| 81 | Gyeonggi | Bucheon-si | 25.39 | 47.33 | 25.33 | 36.94 | 126.79 | 37.50 | 16100.35 | 10.28 | 42.60 | 19235.33 | 67.88 |
| 82 | Gyeonggi | Paju-si | 25.17 | 44.49 | 15.57 | 42.82 | 126.81 | 37.85 | 604.50 | 6.99 | 44.53 | 12998.33 | 64.58 |
| 83 | Gyeonggi | Suwon-si | 23.45 | 43.81 | 24.49 | 39.32 | 127.02 | 37.28 | 9514.58 | 7.37 | 57.53 | 20169.67 | 59.81 |
| 84 | Gyeonggi | Seongnam-si | 22.45 | 43.47 | 23.92 | 38.83 | 127.13 | 37.41 | 6891.20 | 8.16 | 63.33 | 23642.33 | 50.71 |
| 85 | Gyeonggi | Gwangju-si | 25.30 | 45.18 | 17.46 | 41.30 | 127.30 | 37.40 | 683.17 | 3.95 | 49.60 | 5953.67 | 62.36 |
| 86 | Gyeonggi | Yeoncheon-gun | 22.87 | 38.11 | 12.38 | 43.48 | 127.03 | 38.09 | 67.35 | 5.31 | 20.63 | 2623.67 | 68.35 |
| 87 | Gyeonggi | Gunpo-si | 24.13 | 45.43 | 22.55 | 40.39 | 126.92 | 37.34 | 7877.22 | 6.30 | 45.10 | 6373.00 | 58.69 |
| 88 | Gyeonggi | Uiwang-si | 24.23 | 43.25 | 22.58 | 39.97 | 126.99 | 37.36 | 2892.65 | 8.60 | 48.17 | 2549.00 | 57.91 |
| 89 | Gyeonggi | Gwangmyeong-si | 24.91 | 45.82 | 24.27 | 38.81 | 126.87 | 37.44 | 9073.70 | 5.00 | 44.70 | 7793.67 | 64.33 |
| 90 | Gyeonggi | Pyeongtaek-si | 28.09 | 48.86 | 17.22 | 42.09 | 127.02 | 37.03 | 977.47 | 6.05 | 48.03 | 12271.33 | 60.27 |
| 91 | Gyeonggi | Dongducheon-si | 23.32 | 40.90 | 16.42 | 43.74 | 127.08 | 37.91 | 1018.88 | 17.81 | 27.07 | 5564.33 | 75.79 |
| 92 | Gyeonggi | Siheung-si | 26.51 | 48.70 | 22.11 | 40.01 | 126.79 | 37.39 | 2948.35 | 6.85 | 51.43 | 9883.67 | 70.82 |
| 93 | Gyeonggi | Gwacheon-si | 23.51 | 44.75 | 23.33 | 39.42 | 127.01 | 37.43 | 1935.33 | 0.98 | 50.47 | 1228.00 | 35.20 |
| 94 | Gyeonggi | Namyangju-si | 23.83 | 41.92 | 17.50 | 41.76 | 127.25 | 37.66 | 1363.82 | 6.36 | 36.40 | 15670.33 | 66.34 |
| 95 | Gyeonggi | Osan-si | 25.10 | 45.09 | 20.39 | 40.98 | 127.05 | 37.16 | 4772.02 | 8.20 | 44.93 | 4353.00 | 65.43 |
| 96 | Gyeonggi | Hanam-si | 21.95 | 43.90 | 21.46 | 39.43 | 127.21 | 37.52 | 1731.43 | 3.36 | 54.53 | 4212.33 | 55.45 |
| 97 | Gyeonggi | Yongin-si | 25.46 | 44.77 | 18.17 | 41.29 | 127.15 | 37.27 | 1601.23 | 5.77 | 61.60 | 10250.67 | 46.48 |
| 98 | Gyeonggi | Icheon-si | 26.75 | 45.32 | 14.96 | 43.19 | 127.48 | 37.21 | 446.25 | 7.22 | 53.60 | 5047.67 | 64.84 |
| 99 | Gyeonggi | Anseong-si | 25.66 | 43.93 | 14.56 | 43.00 | 127.31 | 37.03 | 328.12 | 8.53 | 35.13 | 5652.67 | 65.49 |
| 100 | Gyeonggi | Hwaseong-si | 27.23 | 47.59 | 16.87 | 42.43 | 126.88 | 37.16 | 808.85 | 5.13 | 65.77 | 9374.67 | 58.72 |
| 101 | Gyeonggi | Pocheon-si | 23.34 | 40.81 | 13.76 | 44.01 | 127.25 | 37.97 | 189.10 | 8.48 | 27.97 | 6151.33 | 67.10 |
| 102 | Gyeonggi | Gapyeong-gun | 21.77 | 37.96 | 13.06 | 43.13 | 127.45 | 37.82 | 72.48 | 10.08 | 25.17 | 3582.00 | 65.15 |
| 103 | Gyeonggi | Yangpyeong-gun | 22.98 | 40.58 | 13.43 | 42.87 | 127.58 | 37.52 | 119.68 | 7.70 | 23.23 | 3826.67 | 56.45 |
| 104 | Gyeonggi | Yeoju-si | 25.10 | 42.82 | 13.79 | 43.53 | 127.62 | 37.30 | 181.08 | 12.32 | 33.37 | 4647.33 | 67.77 |
| 105 | Gangwon | Cheorwon-gun | 22.72 | 38.82 | 12.99 | 43.39 | 127.39 | 38.23 | 53.97 | 5.81 | 12.40 | 2008.33 | 71.47 |
| 106 | Gangwon | Yeongwol-gun | 22.51 | 39.68 | 13.20 | 42.89 | 128.50 | 37.20 | 35.62 | 6.75 | 20.97 | 1496.33 | 76.06 |
| 107 | Gangwon | Chuncheon-si | 21.37 | 39.69 | 14.42 | 43.27 | 127.74 | 37.89 | 247.08 | 11.12 | 28.23 | 13538.00 | 60.33 |
| 108 | Gangwon | Wonju-si | 23.51 | 40.55 | 14.81 | 42.81 | 127.93 | 37.31 | 376.82 | 10.36 | 26.93 | 13032.33 | 65.02 |
| 109 | Gangwon | Gangneung-si | 19.38 | 34.76 | 13.44 | 43.33 | 128.83 | 37.71 | 207.68 | 9.89 | 21.00 | 9563.67 | 69.73 |
| 110 | Gangwon | Donghae-si | 18.23 | 33.25 | 14.59 | 42.36 | 129.06 | 37.50 | 524.07 | 11.46 | 20.40 | 3993.00 | 71.83 |
| 111 | Gangwon | Taebaek-si | 20.65 | 36.13 | 13.36 | 44.17 | 128.98 | 37.17 | 159.98 | 9.21 | 27.63 | 2178.33 | 65.65 |
| 112 | Gangwon | Sokcho-si | 20.16 | 36.92 | 15.34 | 42.74 | 128.52 | 38.17 | 783.65 | 7.49 | 23.67 | 4605.33 | 71.27 |
| 113 | Gangwon | Samcheok-si | 18.25 | 33.80 | 13.18 | 43.57 | 129.12 | 37.27 | 60.45 | 4.66 | 18.77 | 3435.33 | 76.50 |
| 114 | Gangwon | Hongcheon-gun | 22.45 | 39.35 | 13.19 | 43.14 | 128.08 | 37.74 | 38.55 | 8.08 | 17.97 | 2332.00 | 70.73 |
| 115 | Gangwon | Hoengseong-gun | 22.56 | 39.19 | 13.01 | 42.72 | 128.08 | 37.51 | 45.28 | 3.68 | 17.70 | 1743.00 | 70.92 |
| 116 | Gangwon | Pyeongchang-gun | 21.79 | 37.73 | 12.99 | 43.29 | 128.49 | 37.55 | 29.75 | 3.51 | 14.03 | 1375.67 | 69.30 |
| 117 | Gangwon | Jeongseon-gun | 20.59 | 36.83 | 12.78 | 43.41 | 128.74 | 37.38 | 32.42 | 4.97 | 29.37 | 1451.67 | 72.59 |
| 118 | Gangwon | Hwacheon-gun | 21.24 | 36.88 | 12.87 | 43.13 | 127.68 | 38.12 | 28.52 | 1.52 | 14.20 | 1084.33 | 79.35 |
| 119 | Gangwon | Goseong-gun | 19.93 | 36.41 | 12.71 | 43.25 | 128.43 | 38.36 | 45.67 | 6.22 | 14.27 | 1359.00 | 80.28 |
| 120 | Gangwon | Yangyang-gun | 20.20 | 36.69 | 12.87 | 43.35 | 128.60 | 38.00 | 43.82 | 0.78 | 13.00 | 1477.67 | 75.51 |
| 121 | Chungcheongbuk-do | Chungju-si | 23.12 | 40.67 | 13.93 | 43.00 | 127.90 | 37.01 | 211.77 | 9.01 | 19.93 | 8244.00 | 73.86 |
| 122 | Chungcheongbuk-do | Cheongju-si | 24.72 | 41.78 | 15.07 | 43.06 | 127.47 | 36.64 | 885.73 | 8.68 | 37.50 | 25204.00 | 64.75 |
| 123 | Chungcheongbuk-do | Jecheon-si | 22.21 | 38.64 | 13.96 | 42.69 | 128.14 | 37.06 | 155.07 | 12.46 | 20.03 | 6964.00 | 73.09 |
| 124 | Chungcheongbuk-do | Boeun-gun | 22.75 | 39.11 | 12.59 | 44.18 | 127.73 | 36.49 | 58.83 | 14.87 | 9.30 | 1763.33 | 81.31 |
| 125 | Chungcheongbuk-do | Okcheon-gun | 23.24 | 38.25 | 12.61 | 43.95 | 127.66 | 36.32 | 98.25 | 13.54 | 17.77 | 2377.33 | 79.51 |
| 126 | Chungcheongbuk-do | Yeongdong-gun | 22.05 | 38.32 | 12.16 | 44.69 | 127.82 | 36.16 | 59.90 | 10.13 | 16.73 | 2151.67 | 81.31 |
| 127 | Chungcheongbuk-do | Jincheon-gun | 24.79 | 41.91 | 13.25 | 43.46 | 127.44 | 36.87 | 161.32 | 7.31 | 29.40 | 2112.67 | 68.95 |
| 128 | Chungcheongbuk-do | Umseong-gun | 25.17 | 42.83 | 13.30 | 43.47 | 127.62 | 36.97 | 181.83 | 16.06 | 30.40 | 4898.33 | 72.07 |
| 129 | Chungcheongbuk-do | Jeungpyeong-gun | 24.70 | 40.99 | 13.80 | 43.44 | 127.61 | 36.78 | 430.98 | 3.32 | 17.73 | 1317.00 | 71.31 |
| 130 | Chungcheongbuk-do | Danyang-gun | 17.99 | 30.59 | 11.85 | 43.34 | 128.39 | 36.99 | 39.75 | 7.60 | 21.80 | 1530.00 | 79.36 |
| 131 | Chungcheongbuk-do | Gwoisan-gun | 21.31 | 36.00 | 12.27 | 44.06 | 127.83 | 36.77 | 45.35 | 9.81 | 13.50 | 1916.00 | 78.66 |
| 132 | Chungcheongnam-do | Seocheon-gun | 23.25 | 39.74 | 11.79 | 45.29 | 126.71 | 36.10 | 161.23 | 15.55 | 12.73 | 2516.67 | 81.05 |
| 133 | Chungcheongnam-do | Cheonan-si | 25.71 | 43.21 | 15.55 | 43.51 | 127.21 | 36.80 | 934.48 | 10.41 | 46.00 | 13977.00 | 63.33 |
| 134 | Chungcheongnam-do | Asan-si | 25.97 | 44.47 | 14.84 | 43.46 | 126.98 | 36.80 | 533.85 | 8.39 | 45.00 | 8817.33 | 66.70 |
| 135 | Chungcheongnam-do | Gongju-si | 23.22 | 39.96 | 12.68 | 44.43 | 127.08 | 36.48 | 131.73 | 18.91 | 18.23 | 4976.67 | 72.62 |
| 136 | Chungcheongnam-do | Nonsan-si | 22.35 | 40.43 | 12.98 | 44.41 | 127.16 | 36.19 | 225.98 | 18.51 | 14.17 | 7309.67 | 77.70 |
| 137 | Chungcheongnam-do | Boryung-si | 22.78 | 38.82 | 12.53 | 45.15 | 126.62 | 36.32 | 184.13 | 7.82 | 20.90 | 4925.67 | 76.22 |
| 138 | Chungcheongnam-do | Seosan-si | 22.35 | 40.50 | 13.34 | 44.65 | 126.47 | 36.78 | 224.77 | 4.72 | 35.97 | 3786.33 | 71.17 |
| 139 | Chungcheongnam-do | Gyeryong-si | 22.21 | 37.87 | 13.69 | 44.47 | 127.24 | 36.29 | 687.27 | 4.62 | 24.23 | 661.67 | 60.65 |
| 140 | Chungcheongnam-do | Geumsan-gun | 21.84 | 38.39 | 11.91 | 44.22 | 127.48 | 36.12 | 95.87 | 9.46 | 18.80 | 2226.00 | 80.40 |
| 141 | Chungcheongnam-do | Buyeo-gun | 24.23 | 39.38 | 11.65 | 45.02 | 126.86 | 36.24 | 115.55 | 12.11 | 12.97 | 3614.00 | 80.68 |
| 142 | Chungcheongnam-do | Cheongyang-gun | 24.05 | 39.44 | 11.65 | 45.00 | 126.86 | 36.43 | 67.48 | 5.78 | 16.70 | 1060.33 | 80.03 |
| 143 | Chungcheongnam-do | Hongseong-gun | 24.66 | 39.59 | 12.28 | 44.40 | 126.63 | 36.57 | 207.45 | 11.74 | 19.97 | 3477.00 | 75.12 |
| 144 | Chungcheongnam-do | Yesan-gun | 24.77 | 41.29 | 12.38 | 44.40 | 126.79 | 36.67 | 155.88 | 5.31 | 14.47 | 2978.00 | 73.50 |
| 145 | Chungcheongnam-do | Taean-gun | 22.29 | 38.57 | 12.03 | 43.69 | 126.26 | 36.77 | 122.40 | 5.58 | 19.93 | 2227.00 | 73.18 |
| 146 | Chungcheongnam-do | Dangjin-si | 24.78 | 44.64 | 13.85 | 43.95 | 126.66 | 36.89 | 228.53 | 5.46 | 33.53 | 3186.33 | 63.82 |
| 147 | Jeollabuk-do | Gunsan-si | 24.24 | 40.91 | 14.03 | 43.82 | 126.76 | 35.96 | 702.20 | 11.24 | 24.47 | 14671.33 | 73.91 |
| 148 | Jeollabuk-do | Jeonju-si | 24.26 | 41.31 | 16.38 | 43.49 | 127.12 | 35.83 | 3161.10 | 18.54 | 31.27 | 29777.33 | 63.05 |
| 149 | Jeollabuk-do | Wanju-gun | 23.93 | 39.90 | 12.04 | 44.90 | 127.23 | 35.92 | 109.43 | 16.17 | 25.87 | 5007.00 | 78.72 |
| 150 | Jeollabuk-do | Iksan-si | 26.24 | 43.15 | 13.72 | 44.27 | 126.99 | 36.02 | 601.90 | 12.67 | 19.47 | 17674.33 | 75.20 |
| 151 | Jeollabuk-do | Jeongeup-si | 23.43 | 38.34 | 12.28 | 45.32 | 126.91 | 35.60 | 169.95 | 14.17 | 13.80 | 7963.33 | 81.65 |
| 152 | Jeollabuk-do | Namwon-si | 21.17 | 36.90 | 12.00 | 45.31 | 127.44 | 35.42 | 114.22 | 14.39 | 11.67 | 6289.67 | 81.24 |
| 153 | Jeollabuk-do | Gimje-si | 25.05 | 42.09 | 12.96 | 45.10 | 126.91 | 35.80 | 166.13 | 27.14 | 13.70 | 7399.67 | 81.97 |
| 154 | Jeollabuk-do | Jinan-gun | 22.19 | 37.96 | 11.84 | 44.33 | 127.43 | 35.83 | 33.97 | 8.42 | 13.40 | 1457.67 | 83.62 |
| 155 | Jeollabuk-do | Muju-gun | 21.82 | 36.92 | 12.32 | 44.56 | 127.72 | 35.94 | 40.13 | 3.08 | 18.70 | 1268.33 | 84.07 |
| 156 | Jeollabuk-do | Imshil-gun | 22.65 | 38.69 | 11.48 | 44.32 | 127.24 | 35.60 | 50.52 | 7.42 | 13.83 | 1777.67 | 84.34 |
| 157 | Jeollabuk-do | Sunchang-gun | 23.04 | 38.46 | 11.22 | 44.74 | 127.09 | 35.43 | 60.62 | 16.52 | 14.37 | 1469.67 | 83.88 |
| 158 | Jeollabuk-do | Buan-gun | 23.80 | 39.85 | 11.92 | 46.47 | 126.66 | 35.67 | 117.80 | 14.46 | 15.40 | 3191.67 | 82.01 |
| 159 | Jeollanam-do | Mokpo-si | 19.03 | 35.44 | 16.30 | 41.68 | 126.41 | 34.81 | 4745.75 | 21.97 | 23.03 | 13939.67 | 76.20 |
| 160 | Jeollanam-do | Yeosu-si | 17.46 | 31.92 | 14.02 | 41.99 | 127.65 | 34.77 | 574.63 | 11.70 | 34.73 | 11427.67 | 77.76 |
| 161 | Jeollanam-do | Suncheon-si | 19.08 | 33.02 | 12.72 | 44.06 | 127.39 | 34.99 | 303.35 | 14.95 | 26.27 | 8067.33 | 71.89 |
| 162 | Jeollanam-do | Naju-si | 21.02 | 34.68 | 12.21 | 44.96 | 126.72 | 34.99 | 152.65 | 26.28 | 21.27 | 5028.00 | 81.17 |
| 163 | Jeollanam-do | Gwangyang-si | 18.65 | 34.96 | 12.87 | 43.52 | 127.65 | 35.03 | 331.23 | 6.19 | 35.20 | 3906.00 | 73.77 |
| 164 | Jeollanam-do | Damyang-gun | 22.53 | 37.77 | 11.33 | 43.74 | 127.00 | 35.29 | 104.10 | 23.48 | 17.37 | 2198.33 | 78.68 |
| 165 | Jeollanam-do | Gokseong-gun | 20.63 | 35.63 | 11.67 | 44.22 | 127.27 | 35.21 | 56.30 | 12.99 | 15.30 | 1718.00 | 84.85 |
| 166 | Jeollanam-do | Goheung-gun | 19.45 | 33.69 | 11.51 | 43.28 | 127.33 | 34.62 | 88.05 | 14.88 | 12.53 | 4091.00 | 89.48 |
| 167 | Jeollanam-do | Boseong-gun | 21.65 | 35.57 | 11.18 | 43.70 | 127.16 | 34.81 | 69.57 | 22.10 | 13.10 | 2475.67 | 84.84 |
| 168 | Jeollanam-do | Jangheung-gun | 20.04 | 35.08 | 11.14 | 44.22 | 126.92 | 34.67 | 68.45 | 8.30 | 11.67 | 2586.67 | 86.30 |
| 169 | Jeollanam-do | Gangjin-gun | 21.65 | 36.48 | 11.04 | 44.43 | 126.77 | 34.62 | 79.32 | 9.24 | 11.20 | 2100.00 | 83.93 |
| 170 | Jeollanam-do | Haenam-gun | 21.84 | 35.21 | 11.17 | 44.78 | 126.53 | 34.54 | 76.17 | 15.49 | 16.27 | 4191.00 | 85.52 |
| 171 | Jeollanam-do | Yeongam-gun | 21.83 | 34.93 | 11.34 | 44.71 | 126.64 | 34.80 | 97.53 | 12.94 | 13.37 | 2235.67 | 82.68 |
| 172 | Jeollanam-do | Muan-gun | 22.03 | 35.86 | 11.53 | 44.84 | 126.43 | 34.95 | 176.88 | 9.68 | 19.60 | 2752.00 | 82.17 |
| 173 | Jeollanam-do | Hampyeong-gun | 22.26 | 36.22 | 11.01 | 45.46 | 126.54 | 35.11 | 90.03 | 16.67 | 10.70 | 1769.33 | 85.86 |
| 174 | Jeollanam-do | Yeonggwang-gun | 22.69 | 37.45 | 11.41 | 46.09 | 126.46 | 35.27 | 119.47 | 15.64 | 15.70 | 3136.00 | 85.18 |
| 175 | Jeollanam-do | Jangseong-gun | 22.04 | 37.18 | 11.28 | 45.84 | 126.77 | 35.33 | 89.23 | 10.75 | 19.13 | 2406.33 | 80.41 |
| 176 | Jeollanam-do | Jindo-gun | 21.29 | 35.12 | 10.92 | 43.94 | 126.25 | 34.46 | 74.63 | 4.51 | 12.67 | 2037.33 | 87.36 |
| 177 | Jeollanam-do | Hwansun-gun | 19.77 | 33.70 | 11.48 | 43.88 | 127.04 | 35.01 | 85.40 | 47.06 | 21.13 | 3190.33 | 79.15 |
| 178 | Jeollanam-do | Wando-gun | 19.83 | 33.65 | 11.95 | 42.19 | 126.70 | 34.34 | 134.75 | 2.32 | 12.23 | 2538.33 | 89.88 |
| 179 | Jeollanam-do | Gurye-gun | 20.47 | 35.74 | 11.49 | 44.57 | 127.51 | 35.23 | 61.48 | 16.78 | 9.03 | 1423.33 | 82.76 |
| 180 | Gyeongsangbuk-do | Gumi-si | 21.41 | 39.57 | 13.95 | 44.95 | 128.36 | 36.17 | 679.80 | 6.57 | 45.33 | 8819.33 | 70.79 |
| 181 | Gyeongsangbuk-do | Pohang-si | 19.74 | 35.91 | 13.97 | 44.25 | 129.34 | 36.06 | 458.97 | 12.96 | 34.00 | 20353.33 | 71.22 |
| 182 | Gyeongsangbuk-do | Gyeongju-si | 20.95 | 35.94 | 13.03 | 44.22 | 129.24 | 35.82 | 198.00 | 12.14 | 27.97 | 9602.67 | 70.99 |
| 183 | Gyeongsangbuk-do | Gimcheon-si | 22.02 | 38.82 | 12.67 | 45.74 | 128.08 | 36.06 | 136.22 | 10.97 | 28.23 | 6545.33 | 75.11 |
| 184 | Gyeongsangbuk-do | Andong-si | 20.48 | 36.00 | 12.77 | 44.25 | 128.78 | 36.58 | 110.78 | 22.31 | 14.50 | 8701.33 | 75.87 |
| 185 | Gyeongsangbuk-do | Yeongju-si | 22.87 | 39.28 | 12.78 | 44.56 | 128.60 | 36.87 | 166.87 | 14.69 | 19.47 | 6193.33 | 75.85 |
| 186 | Gyeongsangbuk-do | Yeongcheon-si | 20.99 | 37.05 | 12.67 | 44.42 | 128.95 | 36.01 | 110.30 | 13.77 | 18.07 | 6053.00 | 78.66 |
| 187 | Gyeongsangbuk-do | Sangju-si | 22.07 | 38.05 | 12.92 | 44.99 | 128.07 | 36.43 | 82.25 | 8.58 | 14.37 | 4341.67 | 80.49 |
| 188 | Gyeongsangbuk-do | Mungyeong-si | 21.06 | 36.71 | 13.50 | 44.25 | 128.15 | 36.69 | 83.13 | 12.24 | 20.83 | 3243.67 | 75.64 |
| 189 | Gyeongsangbuk-do | Gyeongsan-si | 22.22 | 37.82 | 13.91 | 43.35 | 128.81 | 35.83 | 610.32 | 10.52 | 32.17 | 8887.67 | 69.00 |
| 190 | Gyeongsangbuk-do | Uiseong-gun | 20.78 | 36.41 | 12.17 | 44.53 | 128.62 | 36.36 | 47.45 | 17.98 | 14.63 | 2889.00 | 85.50 |
| 191 | Gyeongsangbuk-do | Yeongyang-gun | 19.93 | 36.26 | 12.61 | 44.28 | 129.15 | 36.69 | 22.18 | 2.35 | 12.37 | 1219.00 | 85.21 |
| 192 | Gyeongsangbuk-do | Yeongdeok-gun | 19.64 | 34.23 | 12.52 | 44.03 | 129.32 | 36.48 | 53.75 | 6.45 | 12.77 | 2300.33 | 84.55 |
| 193 | Gyeongsangbuk-do | Cheongdo-gun | 21.14 | 35.24 | 12.25 | 44.24 | 128.79 | 35.67 | 63.25 | 23.96 | 15.77 | 2106.67 | 76.56 |
| 194 | Gyeongsangbuk-do | Goreong-gun | 19.69 | 35.15 | 11.94 | 44.58 | 128.31 | 35.73 | 91.07 | 6.74 | 19.67 | 1756.67 | 80.12 |
| 195 | Gyeongsangbuk-do | Chilgok-gun | 21.47 | 38.95 | 13.67 | 44.22 | 128.47 | 36.01 | 269.02 | 10.85 | 27.97 | 4238.33 | 72.19 |
| 196 | Gyeongsangbuk-do | Yecheon-gun | 21.74 | 36.58 | 11.84 | 44.74 | 128.43 | 36.65 | 69.08 | 9.27 | 12.23 | 2432.33 | 81.98 |
| 197 | Gyeongsangbuk-do | Bonghwa-gun | 19.58 | 35.92 | 12.53 | 43.89 | 128.92 | 36.93 | 28.20 | 5.55 | 9.63 | 1668.67 | 84.22 |
| 198 | Gyeongsangbuk-do | Uljin-gun | 19.56 | 35.32 | 13.05 | 43.37 | 129.32 | 36.90 | 52.48 | 5.07 | 17.03 | 2689.33 | 83.26 |
| 199 | Gyeongsangnam-do | Yangsan-si | 21.59 | 37.82 | 14.68 | 44.50 | 129.04 | 35.40 | 593.07 | 15.67 | 42.57 | 10028.00 | 71.79 |
| 200 | Gyeongsangnam-do | Gimhae-si | 20.67 | 36.65 | 15.44 | 43.90 | 128.85 | 35.27 | 1125.53 | 13.73 | 41.27 | 16925.67 | 68.37 |
| 201 | Gyeongsangnam-do | Changwon-si | 20.36 | 37.57 | 15.79 | 43.66 | 128.66 | 35.27 | 1445.53 | 13.11 | 41.37 | 27898.33 | 64.35 |
| 202 | Gyeongsangnam-do | Jinju-si | 20.00 | 36.26 | 13.39 | 44.96 | 128.13 | 35.20 | 477.18 | 12.66 | 33.90 | 12852.33 | 68.94 |
| 203 | Gyeongsangnam-do | Tongyeong-si | 18.89 | 34.73 | 14.66 | 43.25 | 128.40 | 34.90 | 581.90 | 11.00 | 21.47 | 6165.67 | 77.16 |
| 204 | Gyeongsangnam-do | Sacheon-si | 18.99 | 34.65 | 13.20 | 44.69 | 128.04 | 35.05 | 289.82 | 18.11 | 22.83 | 5261.33 | 78.13 |
| 205 | Gyeongsangnam-do | Miryang-si | 20.76 | 36.64 | 13.03 | 44.53 | 128.79 | 35.50 | 135.75 | 14.29 | 19.07 | 5427.00 | 72.14 |
| 206 | Gyeongsangnam-do | Geoje-si | 19.40 | 35.21 | 14.34 | 43.68 | 128.63 | 34.86 | 610.43 | 6.77 | 36.13 | 4328.00 | 65.89 |
| 207 | Gyeongsangnam-do | Uiryeong-gun | 21.07 | 35.71 | 12.01 | 44.85 | 128.28 | 35.39 | 60.62 | 14.71 | 16.77 | 1483.67 | 85.25 |
| 208 | Gyeongsangnam-do | Haman-gun | 21.04 | 36.98 | 12.47 | 44.30 | 128.43 | 35.29 | 163.55 | 14.28 | 25.50 | 2560.00 | 76.55 |
| 209 | Gyeongsangnam-do | Changnyeong-gun | 21.35 | 35.98 | 12.45 | 44.28 | 128.50 | 35.51 | 118.95 | 24.55 | 17.87 | 2889.00 | 81.56 |
| 210 | Gyeongsangnam-do | Goseong-gun | 19.88 | 37.07 | 12.13 | 44.28 | 128.29 | 35.01 | 108.32 | 13.08 | 17.37 | 2648.00 | 79.78 |
| 211 | Gyeongsangnam-do | Namhae-gun | 18.89 | 36.04 | 11.97 | 42.92 | 127.93 | 34.81 | 132.07 | 4.70 | 16.53 | 2131.33 | 85.34 |
| 212 | Gyeongsangnam-do | Hadong-gun | 19.89 | 35.64 | 11.22 | 44.56 | 127.78 | 35.14 | 75.02 | 11.09 | 14.83 | 2488.00 | 83.23 |
| 213 | Gyeongsangnam-do | Sancheong-gun | 20.47 | 36.32 | 11.53 | 45.25 | 127.89 | 35.37 | 45.15 | 2.43 | 14.40 | 1911.67 | 81.87 |
| 214 | Gyeongsangnam-do | Geochang-gun | 21.02 | 38.34 | 11.98 | 44.75 | 127.91 | 35.73 | 78.67 | 8.11 | 19.67 | 3089.00 | 80.52 |
| 215 | Gyeongsangnam-do | Hapcheon-gun | 20.38 | 36.80 | 11.91 | 44.77 | 128.14 | 35.57 | 50.63 | 13.09 | 13.20 | 2388.33 | 85.87 |
| 216 | Gyeongsangnam-do | Hamyang-gun | 20.28 | 35.38 | 11.71 | 44.82 | 127.72 | 35.55 | 55.98 | 2.53 | 16.77 | 2081.67 | 82.70 |
| 217 | Sejong | Sejong | 24.11 | 42.39 | 14.96 | 44.02 | 127.22 | 36.53 | 363.66 | 3.68 | 66.97 | 4965.67 | 58.89 |

**Table A.2. Descriptive summary of air pollution concentrations (NO_2_, and O_3_).**

|  | Mean | SD | Min | Max | IQR^a^ |
| --- | --- | --- | --- | --- | --- |
| 2016 |  |  |  |  |  |
| NO_2_ | 17.28 | 5.17 | 11.68 | 29.07 | 7.17 |
| O_3_ | 41.91 | 2.66 | 34.04 | 46.85 | 3.50 |
| 2018 |  |  |  |  |  |
| NO_2_ | 16.42 | 4.86 | 11.07 | 27.65 | 7.30 |
| O_3_ | 41.94 | 2.98 | 33.83 | 46.57 | 4.27 |
| 2020 |  |  |  |  |  |
| NO_2_ | 15.11 | 4.46 | 9.62 | 25.16 | 7.31 |
| O_3_ | 42.91 | 2.75 | 35.77 | 47.09 | 3.74 |

^a^ Interquartile range derived by Q3-Q1.

NO_2_: nitrogen dioxide; O_3_: ozone.

**Table A.3. Associations between long-term air pollution exposure (per 10** $\boldsymbol{\mu g}\mathbf{/}\boldsymbol{m}^{\boldsymbol{3}}$ **increment) and changes in CES-D 10 score by linear mixed models.**

|  | Estimates (95% CI) | |
| --- | --- | --- |
|  | PM_2.5_ | PM_10_ |
| Crude model | 0.43 (0.29, 0.57) | 0.22 (0.15, 0.30) |
| Adjusted model^a^ |  |  |
| Single pollutant (main analysis) | 0.62 (0.48, 0.77) | 0.35 (0.27, 0.44) |
| Two-pollutant (plus NO_2_) | 0.61 (0.44, 0.77) | 0.37 (0.27, 0.47) |
| Two-pollutant (plus O_3_) | 0.65 (0.50, 0.80) | 0.38 (0.29, 0.47) |
| One-year average concentrations before  panel examination period^b^ | 0.72 (0.56, 0.87) | 0.42 (0.33, 0.52) |

^a^ Adjusted for baseline CES-D 10 score, sex, age group, current smoking, current drinking, education attainment, marital status, social contact, self-reported health status, exercise, private medical insurance, living area, longitude, latitude, and the interaction term of longitude and latitude, population density, the number of beds in hospitals per 1,000 persons, the number of national basic livelihood beneficiaries, the independent rate of finance of local government, and the proportion of basic pension beneficiaries.

^b^ Average concentration from September of the previous year to August of the current year in 2016, 2018, and August of the previous year to July of the current year in 2020.

CI: confidence interval; PM_2.5_: particulate matter with an aerodynamic diameter ≤ 2.5 µm; PM_10_: particulate matter with an aerodynamic diameter ≤ 10 µm; NO_2_: nitrogen dioxide; O_3_: ozone.

**Table A.4. Associations between long-term air pollution exposure (per 10** $\boldsymbol{\mu g}\mathbf{/}\boldsymbol{m}^{\boldsymbol{3}}$ **increment) and changes in CES-D 10 score by generalized linear mixed models.**

|  | Odds ratio (95% CI) | |
| --- | --- | --- |
|  | PM_2.5_ | PM_10_ |
| Crude model | 1.27 (1.13, 1.43) | 1.14 (1.07, 1.22) |
| Adjusted model^a^ |  |  |
| Single pollutant (main analysis) | 1.37 (1.20, 1.57) | 1.19 (1.10, 1.29) |
| Two-pollutant (plus NO_2_) | 1.43 (1.24, 1.65) | 1.25 (1.14, 1.37) |
| Two-pollutant (plus O_3_) | 1.44 (1.26, 1.65) | 1.24 (1.14, 1.34) |
| One-year average concentrations before  panel examination period^b^ | 1.47 (1.27, 1.70) | 1.25 (1.14, 1.36) |

^a^ Adjusted for baseline depression status, sex, age group, current smoking, current drinking, education attainment, marital status, social contact, self-reported health status, exercise, private medical insurance, living area, longitude, latitude, and the interaction term of longitude and latitude, population density, the number of beds in hospitals per 1,000 persons, the number of national basic livelihood beneficiaries, the independent rate of finance of local government, and the proportion of basic pension beneficiaries.

^b^ Average concentration from September of the previous year to August of the current year in 2016, 2018, and August of the previous year to July of the current year in 2020.

Note: We estimated the odds ratios from the generalized linear mixed model (GLMM) with a binomial distribution, logit link, and random intercept for each subject. Depression was defined as having a CES-D 10 score of 20 points or over.

CI: confidence interval; PM_2.5_: particulate matter with an aerodynamic diameter ≤ 2.5 µm; PM_10_: particulate matter with an aerodynamic diameter ≤ 10 µm; NO_2_: nitrogen dioxide; O_3_: ozone.

**Table A.5. Summary of the previous studies on the long-term association between particulate matters and depression.**

| **Study** | **Region,**  **N, Age** | **Study year** | **Study design/analysis model** | **number of measurement locations** | **Observed air pollution/ modeled air pollution** | **Concentrations**  **(μg/m^3^)** | **Outcome variable** | **Long-term effect size (reported)** | **Effect size per 10 μg/m^3^ increase**  **(converted)** |
| --- | --- | --- | --- | --- | --- | --- | --- | --- | --- |
| Lamichhane et al., 2021 | Seoul, Korea,  N=1481 (pregnant women), 32.9±3.6 | 2008-2015 | Cross-sectional study from cohort data, poisson regression | 40 monitoring sites | Observed air pollution  (Korean Ministry of Environment) | PM_2.5_: 26.6 PM_10_: 49.2 | CES-D 10 | IQR (PM_2.5_: 6.9, PM_10_: 8.5) increase in PM during the second trimester (14-27 weeks),  RR (95% CI) - PM_2.5_: 1.15 (1.04-1.27), - PM_10_: 1.13 (1.04-1.23) | RR (95% CI) - PM_2.5_: 1.22 (1.06-1.41), - PM_10_: 1.15 (1.05-1.28) |
| Kim et al., 2016 | Seoul, Korea, N=27270, 15-79 | 2007-2010 | Cohort study, Cox proportional hazard model | 25 districts in Seoul (27 monitoring sites) | Observed air pollution (Research institute of public health and environment) | PM_2.5_: 26.7 | Diagnosis of major depressive disorder (ICD-10 code F32.x) | 10-unit increase in 12-month moving average concentration of  PM_2.5_,  HR (95% CI)  - PM_2.5_: 1.47 (1.14-1.90) | HR (95% CI)  - PM_2.5_: 1.47 (1.14-1.90) |
| Kim et al., 2021 | South Korea,  N=127945  (depression: 25589, control: 102356),  Over 40 years | 2002-2013 | Nested case-control study, conditional logistic regression | 273 monitoring sites | Observed air pollution  (Korean Ministry of Environment) | PM_10_: 53.3 | Diagnosis for depression  (ICD-10 code F31-33) | 10-unit increase in 365-days average concentration of  PM_10_,  HR (95% CI)  - PM_10_: 1.03 (1.00-1.05) | HR (95% CI)  - PM_10_: 1.03 (1.00-1.05) |
| Zhang et al., 2019 | South Korea,  N=123045,  39.4±6.8 | 2011-2015 | Cohort study, Cox proportional hazard model | Monitoring sites in Seoul and Gyeonggi-do | Modeled air pollution | PM_2.5_: 24.3±1.3  PM_10_: 50.6±4.5 | CES-D | 10-unit increase in 12-month average concentrations of  PM,  HR (95% CI)  - PM_2.5_: 1.01 (0.83-1.22)  - PM_10_: 1.11 (1.06-1.16) | HR (95% CI)  - PM_2.5_: 1.01 (0.83-1.22)  - PM_10_: 1.11 (1.06-1.16) |
| Wang et al., 2020 | China,  N=24623, 67.2±6.3 | 2011, 2013, 2015 | Longitudinal study, multilevel linear models | 150 city-level divisions | Modeled air pollution | PM_2.5_: 33.1 | CES-D 10 | logarithm of annual average concentration of PM_2.5_,  Coef (SE)  PM_2.5_: 0.57 (0.11) |  |
| Wang et al., 2019 | China,  N=20861, 44.83±14.61 | 2016 | Cross-sectional study, multilevel regressions | 158 prefecture-level administrative divisions (1613 monitoring stations) | Observed air pollution  (Airborne [Fine Particulate Matter](https://www.sciencedirect.com/topics/medicine-and-dentistry/fine-particulate-matter) and Air Quality Index website) | PM_2.5_: 70.4±37.8 | CES-D | logarithm of average annual concentration of  PM_2.5_,  Coef (SE)  - PM_2.5_: 2.54 (0.34) |  |
| Xue et al., 2021 | China, N=15954, 60.5±9.3 | 2011, 2013, 2015 | Longitudinal study, difference-in-difference (DID) analysis and linear mixed-effect model (LMM) | 150 city-level divisions | Modeled air pollution | PM_2.5_: 58.2±19.8 | CES-D 10 | 10-unit increase in annual average concentration of PM_2.5_,  Excess risk (95% CI)  - DID: 4.14% (0.41-8.00%)  - LMM: 3.63% (2.00-5.27%) | Excess risk (95% CI)  - DID: 4.14% (0.41-8.00%)  - LMM: 3.63% (2.00-5.27%) |
| Wei et al., 2022 | China,  N=30712,  62.2±11.3 | 2016-2020 | Cohort study,  Time-varying Cox regression | 377 sites in east China | Modeled air pollution | PM_2.5_: 34.6 PM_10_: 51.0 | Diagnosis for depression  (ICD-10 code F32) | IQR (PM_2.5_: 5.8, PM_10_: 11.3) increase in one-year average concentrations of PM,  HR (95% CI) - PM_2.5_: 1.44 (1.29-1.60), - PM_10_: 1.41 (1.27-1.57) | HR (95% CI) - PM_2.5_: 1.88 (1.56-2.26), - PM_10_: 1.35 (1.23-1.49) |
| Wang et al., 2022 | Taiwan, N=1956, 73.4±4.9 | 1996, 1999, 2003, 2007 | Longitudinal study, generalized linear mixed model | 75 monitoring sites | Observed air pollution  (Taiwan's Environmental Protection Administration) | PM_10_: 65.8±15.7 | CES-D 10 | IQR (PM_10_: 17.5) increase in annual concentration of PM_10_,  OR (95% CI)  - PM_10_: 0.99 (0.91-1.09) | OR (95% CI)  - PM_10_: 0.99 (0.95-1.05) |
| Kioumourtzoglou et al., 2017 | US, N=41844 (Women), 66.6±7.6 | 1996-2008 | Longitudinal study, Time-varying Cox regression | 2018 sites | Modeled air pollution | PM2.5:  12.6 ± 2.4 in Northeast  13.9 ± 2.2 in Midwest  12.2 ± 4.7 in West  11.6 ± 2.4 in South | Depression diagnosed by a physician or antidepressant use | 10-unit increase in one-year average concentration of PM_2.5_,  HR (95% CI)  - PM_2.5_: 1.08 (0.97-1.20) | HR (95% CI)  - PM_2.5_: 1.08 (0.97-1.20) |
| Pun et al., 2017 | US, N=4008, 69.3±7.8 in 2005-2006; 72.4±8.1 in 2010-2011 | 2005-2006, 2010-2011 | Longitudinal study, generalized linear mixed models | 894 6-km grids closest to residential addresses | Modeled air pollution | PM_2.5_:  11.1 ± 3.0 in 2005-2006; 8.8 ± 2.3 in 2010-2011 | CES-D 11 | 5-unit increase in 365-days average concentration of  PM_2.5_,  OR (95% CI)  - PM_2.5_: 1.10 (0.93-1.31) | OR (95% CI)  - PM_2.5_: 1.21 (0.86-1.72) |
| Qiu et al., 2023 | US, N=8907422, 73.7 ± 4.8 | 2005-2016 | Longitudinal study, Cox proportional hazard models | Nationwide zip code-level allocation from 1km-grid predictions | Modeled air pollution | PM_2.5_: 9.6±2.7 | Depression identified via an algorithm in Chronic Condition Warehouse (CCW) | 5-unit increase in past-5-years average concentration of PM_2.5_,  HR (95% CI)  - PM_2.5_: 1.02 (1.01-1.03) | HR (95% CI)  - PM_2.5_: 1.04 (1.03-1.06) |
| Altug et al., 2020 | Germany  (elderly women),  N=821, 73.5±3.0 | 2007-2010 | Cross-sectional study from cohort data, multivariable logistic regression | 20 sites | Modeled air pollution | PM_2.5_: 17.4  PM_10_: 26.4 | Self-reported diagnosis of depression,  CESD-R | IQR (PM_2.5_: 1.8, PM_10_: 2.2) increase in annual concentration of PM,  [self-reported diagnosis of depression]  OR (95% CI)  - PM_2.5_: 1.62 (1.06-2.46)  - PM_10_: 1.25 (0.94-1.67);  [CESD-R]  %diff in the mean  - PM_2.5_: 16.2 (5.8-26.5)  - PM_10_: 7.9 (0.4-15.4) | [self-reported diagnosis of depression]  OR (95% CI)  - PM_2.5_: 14.59 (1.38-148.55)  - PM_10_: 2.76 (0.75-3.69);  [CESD-R]  %diff in the mean  - PM_2.5_: 90.2 (32.2-147.2)  - PM_10_: 35.9 (1.82-70.0) |

**Figure A.3. Exposure-response curves of the particulate matters (PM_2.5_ and PM_10_) on depression based on the continuous scale of the CES-D 10 score.** Non-linear curve between PM_2.5_ and CES-D 10 score **(A)**, non-linear curve between PM_10_ and CES-D 10 score **(B)**. The blue lines represent the 10^th^, 25^th^, 75^th^, and 90^th^ percentiles of the PM_2.5_ and PM_10_ concentration distributions, respectively. Exposure-response curves were obtained from the generalized additive models (GAM), after adjusting for sex, age group, current smoking, current drinking, education attainment, marital status, social contact, self-reported health status, exercise, private medical insurance, longitude, latitude, and the interaction term of the longitude and latitude, population density, the number of beds in hospitals per 1,000 persons, the number of national basic livelihood beneficiaries, the independent rate of finance of local government, and the proportion of basic pension beneficiaries. PM_2.5_: particulate matter with an aerodynamic diameter ≤ 2.5 µm; PM_10_: particulate matter with an aerodynamic diameter ≤ 10 µm; CES-D: center for epidemiology studies of depression scale.

**A**


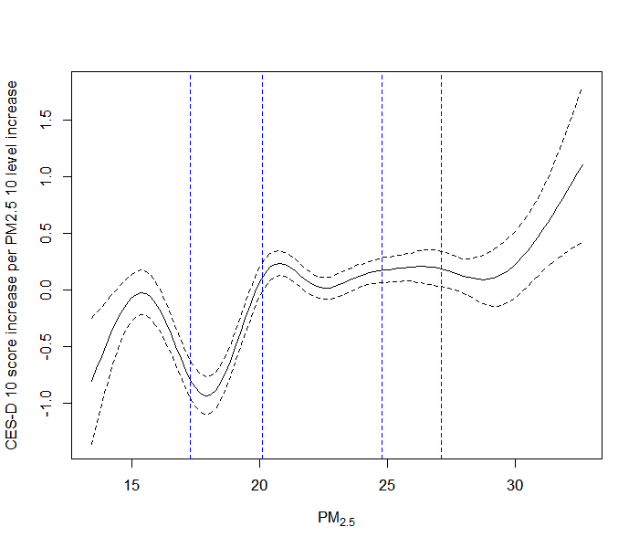


**B**

***
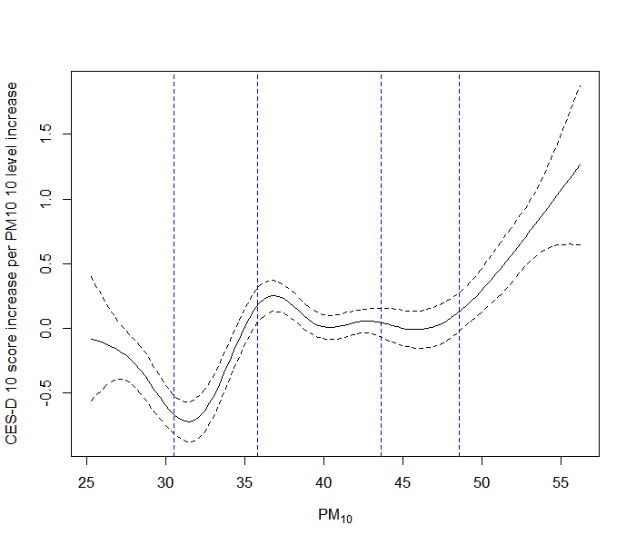
***
